# Regional Patterns and Association Between Obesity and Hypertension in Africa: Evidence From the H3Africa CHAIR Study

**DOI:** 10.1161/HYPERTENSIONAHA.119.14147

**Published:** 2020-03-16

**Authors:** Onoja M. Akpa, Felix Made, Akinlolu Ojo, Bruce Ovbiagele, Dwomoa Adu, Ayesha A. Motala, Bongani M. Mayosi, Sally N. Adebamowo, Mark E. Engel, Bamidele Tayo, Charles Rotimi, Babatunde Salako, Rufus Akinyemi, Mulugeta Gebregziabher, Fred Sarfo, Kolawole Wahab, Godfred Agongo, Marianne Alberts, Stuart A. Ali, Gershim Asiki, Romuald P. Boua, F. Xavier Gómez-Olivé, Felistas Mashinya, Lisa Micklesfield, Shukri F. Mohamed, Engelbert A. Nonterah, Shane A. Norris, Hermann Sorgho, Stephen Tollman, Rulan S. Parekh, Chishala Chishala, Kenneth Ekoru, Salina P. Waddy, Emmanuel Peprah, George A. Mensah, Ken Wiley, Jennifer Troyer, Michèle Ramsay, Mayowa O. Owolabi, as members of the CVD Working Group of the H3Africa Consortium

**Affiliations:** 1 From the Center for Genomic and Precision Medicine, College of Medicine, (O.M.A., B.S., R.A., M.O.O.), University of Ibadan, Ibadan, Nigeria; 2 Department of Epidemiology and Medical Statistics, College of Medicine (O.M.A.), University of Ibadan, Ibadan, Nigeria; 3 Institute of Cardiovascular Diseases, College of Medicine (O.M.A.), University of Ibadan, Ibadan, Nigeria; 4 The Epidemiology and Surveillance Section, National Institute for Occupational Health, National Health Laboratory Services, Gauteng Region, South Africa (F. Made); 5 Sydney Brenner Institute for Molecular Bioscience and Division of Human Genetics, Faculty of Health Sciences, University of the Witwatersrand, Johannesburg, South Africa (O.M.A., F. Made, S.A.A., M.R.); 6 Clinical research and global health initiatives, University of Arizona Health Sciences (A.O.); 7 Department of Neurology, University of California, San Francisco CA, USA (B.O.); 8 School of Medicine and Dentistry, University of Ghana, Accra, Ghana (D.A.); 9 Department of Diabetes and Endocrinology, Nelson R. Mandela School of Medicine, University of KwaZulu-Natal, Durban, South Africa (A.A.M.); 10 Department of Medicine, Groote Schuur Hospital (B.M.M.), University of Cape Town, South Africa; 11 Division of Cardiology, Department of Medicine (M.E.E., C.C.), University of Cape Town, South Africa; 12 Department of Epidemiology and Public Health, Greenebaum Comprehensive Cancer Center, University of Maryland School of Medicine, Baltimore, MD, USA (S.N.A.); 13 Department of Preventive Medicine and Epidemiology, Loyola University Chicago Stritch School of Medicine, Maywood, IL (B.T.); 14 Center for Research on Genomics and Global Health, NHGRI, NIH, Bethesda, MD, USA (C.R.); 15 Department of Public Health Sciences, Medical University of South Carolina, Charleston SC, USA (M.G.); 16 Kwame Nkrumah University of Science and Technology, Kumasi, Ghana (F.S.); 17 Department of Medicine, University of Ilorin, Nigeria (K. Wahab); 18 Navrongo Health Research Centre, Ghana (G. Agongo, E.A.N.); 19 Department of Pathology and Medical Science, School of Health Care Sciences, Faculty of Health Sciences, University of Limpopo, Polokwane, South Africa (M.A., F. Mashinya); 20 African Population and Health Research Center, Nairobi, Kenya (G. Asiki, S.F.M.); 21 Institut de Recherche en Sciences de la Sante, Clinical Research Unit of Nanoro, Burkina Faso (R.P.B., H.S.); 22 MRC/Wits Rural Public Health and Health Transitions Research Unit (Agincourt), School of Public Health, Faculty of Health Sciences, University of the Witwatersrand, Johannesburg, South Africa (F.X.G.-O., S.T.); 23 MRC/Wits Developmental Pathways for Health Research Unit, Faculty of Health Sciences, University of the Witwatersrand, Johannesburg, South Africa (L.M., S.A.N.); 24 Departments of Pediatrics, Medicine and Epidemiology, Hospital for Sick Children, University Health Network and University of Toronto, Canada (R.S.P.); 25 Center for Research on Genomics and Global Health, National Human Genome Research Institute, National Institutes of Health, Bethesda, MD (K.E.); 26 Department of Neurology, Atlanta Veterans Affairs Medical Center, Decatur, GA (S.P.W.); 27 College of Global Public Health, New York University, New York, NY (E.P.); 28 Center for Translation Research and Implementation Science, National Heart, Lung, and Blood Institute, NIH, Bethesda, MD (G.A.M.); 29 Division of Genomic Medicine, National Human Genome Research Institute, Bethesda, MD (K. Wiley); 30 Human Heredity and Health in Africa, Division of Genome Sciences (J.T.), National Institutes of Health, Bethesda, MD.

**Keywords:** Africa, blood pressure, hypertension, obesity, risk factors

## Abstract

Hypertension and obesity are the most important modifiable risk factors for cardiovascular diseases, but their association is not well characterized in Africa. We investigated regional patterns and association of obesity with hypertension among 30 044 continental Africans. We harmonized data on hypertension (defined as previous diagnosis/use of antihypertensive drugs or blood pressure [BP]≥140/90 mmHg/BP≥130/80 mmHg) and obesity from 30 044 individuals in the Cardiovascular H3Africa Innovation Resource across 13 African countries. We analyzed data from population-based controls and the Entire Harmonized Dataset. Age-adjusted and crude proportions of hypertension were compared regionally, across sex, and between hypertension definitions. Logit generalized estimating equation was used to determine the independent association of obesity with hypertension (*P* value <5%). Participants were 56% women; with mean age 48.5±12.0 years. Crude proportions of hypertension (at BP≥140/90 mmHg) were 47.9% (95% CI, 47.4–48.5) for Entire Harmonized Dataset and 42.0% (41.1–42.7) for population-based controls and were significantly higher for the 130/80 mm Hg threshold at 59.3% (58.7–59.9) in population-based controls. The age-adjusted proportion of hypertension at BP≥140/90 mmHg was the highest among men (33.8% [32.1–35.6]), in western Africa (34.7% [33.3–36.2]), and in obese individuals (43.6%; 40.3–47.2). Obesity was independently associated with hypertension in population-based controls (adjusted odds ratio, 2.5 [2.3–2.7]) and odds of hypertension in obesity increased with increasing age from 2.0 (1.7–2.3) in younger age to 8.8 (7.4–10.3) in older age. Hypertension is common across multiple countries in Africa with 11.9% to 51.7% having BP≥140/90 mmHg and 39.5% to 69.4% with BP≥130/80 mmHg. Obese Africans were more than twice as likely to be hypertensive and the odds increased with increasing age.

## Abstract 2

Supplemental Digital Content is available in the text.

Hypertension and obesity are major public health concerns that contribute substantially to the rising global trend in morbidity and premature mortality. The global burden of disease attributable to hypertension has significantly increased from ≈4.5% in 2000^1,2^ to 7% in 2010^3^ and is predicted to rise to as high as 29.2% (28.8%–29.7%) by 2025.^1^ Compared with other WHO regions, Africa has the highest prevalence of hypertension with an overall prevalence of 46% in adults aged 25 years and above.^4^ Obesity is an important risk factor for hypertension and other cardiovascular diseases.^5^ Its prevalence is on the rise globally and current estimates show that 20% to 50% of people living in urban populations in Africa are either overweight or obese.^5^ Thus, obesity and hypertension are 2 of the most important risk factors for morbidity and mortality in Africa. Therefore, there is an urgent need for cross-country research into the underlying risk factors and relationship between these diseases to derive appropriate interventions to reduce their surging impact.

Previous smaller studies have found associations between hypertension and obesity in selected countries in sub-Saharan Africa.^1,2,5^ A study conducted in urban Tanzania showed that elevated body mass index (BMI) was associated with a 10% increase in the odds of having hypertension^5^ while in a study conducted in Enugu metropolis (an urban area) of Nigeria, obesity increased the odds of hypertension by 50%.^6^

However, the relationship between obesity and hypertension has not been examined simultaneously across multiple African countries. Moreover, the extent to which the new lower threshold for diagnosing hypertension^7,8^ will affect the estimates of the burden of hypertension and its association with obesity in African populations is unknown. Analyzing these associations across Africa will provide more robust and generalizable estimates, elucidate regional and country-specific distribution patterns of hypertension and obesity, and clarify the effect size of the relationship between obesity and hypertension throughout the regions. Such data are critical in planning the much-needed intervention strategies and policy decisions to control obesity and hypertension in Africa. In this study, the largest thus far in Africa, we investigated the associations between obesity and hypertension and explored the impact of applying the 2017 definition of hypertension on the demographic and regional patterns of the burden of hypertension in Africa. We utilized data from participants living in 13 African countries who are part of the Cardiovascular H3Africa Innovation Resource (CHAIR).^9^

## Methods

The data, analytic methods, and study materials that support the findings of this study will be made available to other researchers upon reasonable request and approval by the CHAIR group of the H3Africa.

### Source of Data and Data Extraction

We utilized data from 30 044 individuals from 13 African countries that were recruited through 5 H3Africa projects including the Africa Wits-INDEPTH Partnership for Genomic studies (AWI-Gen; focusing on genomic and environmental risk factors for cardiometabolic diseases in Africans)^10^; burden, spectrum and etiology of type 2 diabetes mellitus in sub-Saharan Africa (DM)^11^; H3Africa Kidney Disease Research Network (Kidney)^12^; Genetics of rheumatic heart disease Network (RHDGen)^13^; and Stroke Investigative Research & Educational Network (SIREN).^14^ Data were extracted and harmonized for all study participants recruited before September 30, 2016. Ethics approval was obtained at all clinical centers, hospitals, and universities affiliated with the H3Africa studies. Informed consent was provided by all participants, and ethics approval details are provided in the papers referenced for each of the studies above.

Analyses were limited to age, sex, country of residence, weight, height, history of hypertension, hypertension medication status, systolic and diastolic blood pressures (BPs). Countries of residence were grouped into regions according to the African Union (AU) grouping: East Africa (Kenya, Tanzania and Uganda); Central Africa (Cameroon); North Africa (Sudan); Southern Africa (Mozambique, Namibia, South Africa and Zambia); and West Africa (Burkina Faso, Ghana, Guinea and Nigeria).^15^ Cardiovascular variables measured across studied were harmonized.

### Design of the Participating Studies

The studies in the CHAIR dataset are either cross-sectional population or disease-based or case-control studies (Table S5 in the Data Supplement). The study protocols and recruitment strategies are published. Briefly, AWI-Gen is a population based cross-sectional study of adults aged 40 to 60 years recruited at 6 sites in 4 African countries.^10^ The study on DM is a cross-sectional study comprising a clinic arm of participants with known diabetes mellitus at health facilities and in parallel, from the same geographic area, a population survey arm.^11^ The Kidney Disease Research Network had a case-control study design of those with chronic kidney disease and kidney-disease-free controls from Ghana and Nigeria.^12^ RHDGen is a case-control study with cases of rheumatic heart disease recruited in a hospital-based setting and community-based rheumatic heart disease-free controls.^12^ SIREN is a case-control study with cases of stroke and population based stroke-free participants recruited from similar communities in Nigeria. Participants in SIREN were followed up for 1 year after enrolment, but only baseline data are included in this publication.^14^

### Eligibility Criteria for Being Defined as Population Controls

To be included in the population controls (PC) analyses in the study, individuals were either for a population cross-sectional study or were disease-free participants from other studies, as described below. The population-based study, AWI-Gen recruited population-based controls, without consideration of disease phenotype, from both urban and rural communities.^10^ Kidney disease–free controls were adults recruited from the communities, ambulatory clinics, or hospital wards.^12^ In RHDGen, controls were community-based individuals with no valvular heart diseases by echocardiography.^13^ The SIREN study recruited adult population controls from the communities during community outreach activities. Controls were adults (>18 years) who were unrelated to the cases, with or without cardiovascular risk factors but with no evidence of stroke (Table S5).^14^ All regional comparisons were limited to data from population-based or disease-free controls who were available in 4 of the 5 studies.

### Harmonization Procedures

We obtained the raw individual level data (for each study) on age, sex, country of residence, weight, height, and BP and then calculated obesity (or BMI), and hypertension status. Data from the one population-based cross-sectional study (AWI-Gen) and the disease-free controls recruited across 3 studies (Kidney group, RHDGen, and SIREN) were harmonized and analyzed as the PCs while the combined harmonized dataset with all the participants, including those with stroke, kidney disease, and diabetes mellitus, was analyzed as the entire harmonized data (EHD).

Variables included were limited to baseline data. For phenotypes such as weight and height where units of measurement differed between studies, data were converted to kilograms for weight and meters for height. Details of the harmonization strategies for selected phenotypes in CHAIR are reported in study by Owolabi et al^9^ earlier and summarized in Table S7.

### Definitions of Phenotypes

Demographic characteristics included age (at the time of data collection), participants’ sex and country of residence. Baseline BP was measured 3× for each participant across all studies using appropriate tools and sphygmomanometer cuff sizes according to standard procedures.^16^ An average of at least 2 of the baseline readings per participant was utilized for this analysis.^16^

Hypertension was defined as the presence of one or more of the following conditions: reported diagnosis by a health worker or history of hypertension (in some studies confirmed from medical records), current use of medication for hypertension, having a systolic BP ≥140 mm Hg and/or diastolic BP ≥90 mm Hg.^17^ The data were also analyzed for the 2017 hypertension definition using the 130/80 mm Hg BP threshold.^6^ Overweight was defined as body mass index (BMI: weight in kilograms per height in meters squared) between 25.0 and 29.9 kg/m^2^ while obesity was defined as BMI≥30 kg/m^2^ in line with WHO guidelines.^17^

### Statistical Analysis

Descriptive statistics included mean and SDs for continuous variables; numbers and percentages for categorical variables, overall and stratified by sex. Northern and Central Africa were removed from regional comparisons because they each had data for only one country, with small sample sizes, resulting in potentially distorting results. Regional age-adjusted proportions of hypertension and obesity were estimated using the WHO’s World Standard Population Distribution, based on world average population between 2000 and 2025^18^ and compared between regions having control data for more than one country across studies. The 2-sample independent *t*-test was used to assess average difference of continuous variables and the *z*-test for comparing proportions.

We assessed the sex-specific differences in age, BMI, obesity, and proportion of hypertension across participants’ country of residence, geographic region, and age group. Less than 5% of data on selected phenotypes (like height and weight) were missing completely at random and hence, no imputation was carried out. Analyses were conducted for the EHD and the Population Control Dataset (PC) with hypertension defined at 2 BP thresholds: (≥140/90 and ≥130/80 mmHg BP thresholds). Logit generalized estimating equation accounting for the clustering by country was fitted to assess the association between obesity and hypertension (with age and country of residence as covariates, and in addition, sex was added as a covariate in the analysis of the combined dataset). These covariates were the only data available across all the participant studies study at the time of analysis and were all found to be associated with hypertension in bivariate analyses. The adjusted odds ratio (aOR) and 95% CI were estimated using multivariable logit generalized estimating equation models. All analyses were carried out using R statistical package and IBM SPSS Statistics version 22 (Tibco Software, CA).

## Results

### Participants’ Characteristics

Overall, there were 30 044 participants (Table S1) with a mean±SD age of 49.2±13.4 years for men and 48.8±13.0 years for women. Mean BMI was 26.1±16.3 kg/m^2^ (24.5±16.7 kg/m^2^ for men and 28.2±15.8 kg/m^2^ for women). Demographic parameters were similar between EHD and PC with women being a bit younger in both groups and comprising 57.0% in the EHD (Table S1) and 55.6% in the PC (Table 1).

**Table 1.**
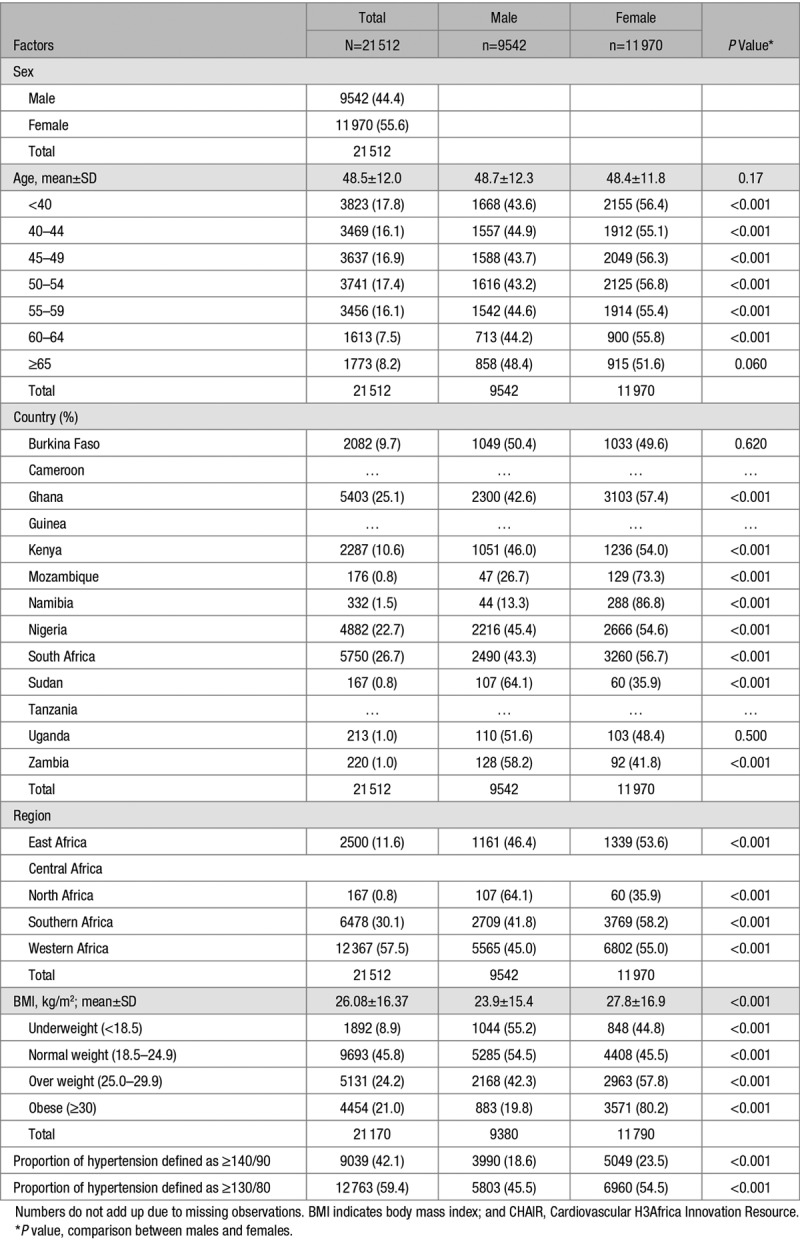
Demographic, Obesity, and Hypertension Information Stratified by Sex and Assessing Significant Sex-Related Differences in the CHAIR Population Controls (PC)

### Burden of Hypertension and Obesity

Overall, 23.3% (10.8% of men and 32.8% of women) were obese in the EHD (Table S1) while 21.1% (9.4% of men and 30.4% for women) were obese in the PC (Table 1). In the EHD, 47.9% (95% CI: 47.4 to 48.5) were hypertensive (Table S2) while 42.0% (41.4–42.7) of the PC had hypertension (Table 2). Age-adjusted proportion of hypertension was 35.1% (34.3–36.0) for the EHD (Table S2) and 32.0% (30.9–33.0) for the PC (Table 2). Men (48.7%; 47.9–49.6) had significantly higher crude proportion of hypertension than women (47.3% [95% CI, 46.5–48.1]) in the EHD, with lower age-adjusted proportions (Table S3). In PC, the crude and age-adjusted proportions of hypertension were lower than the EHD but similar between men (41.8% [40.8–42.8] with age-adjusted: 33.8% [32.1–35.6]) and women (42.2% [41.3–43.1] with age-adjusted: 30.5% [29.2–31.9]; Table 3). The proportions with hypertension and obesity in the EHD across studies are presented in Table S6. Crude hypertension was most common in the SIREN participants (76.9%) and least frequent in the RHDGen (21.4%) study while obesity was more common among participants in the DM study (40.8%; Table S6). Also, as expected, in both the EHD (Table S2) and the PC (Table 2), the proportion of hypertension was substantially higher using the 2017 guidelines compared with the old definition of hypertension.

**Table 2.**
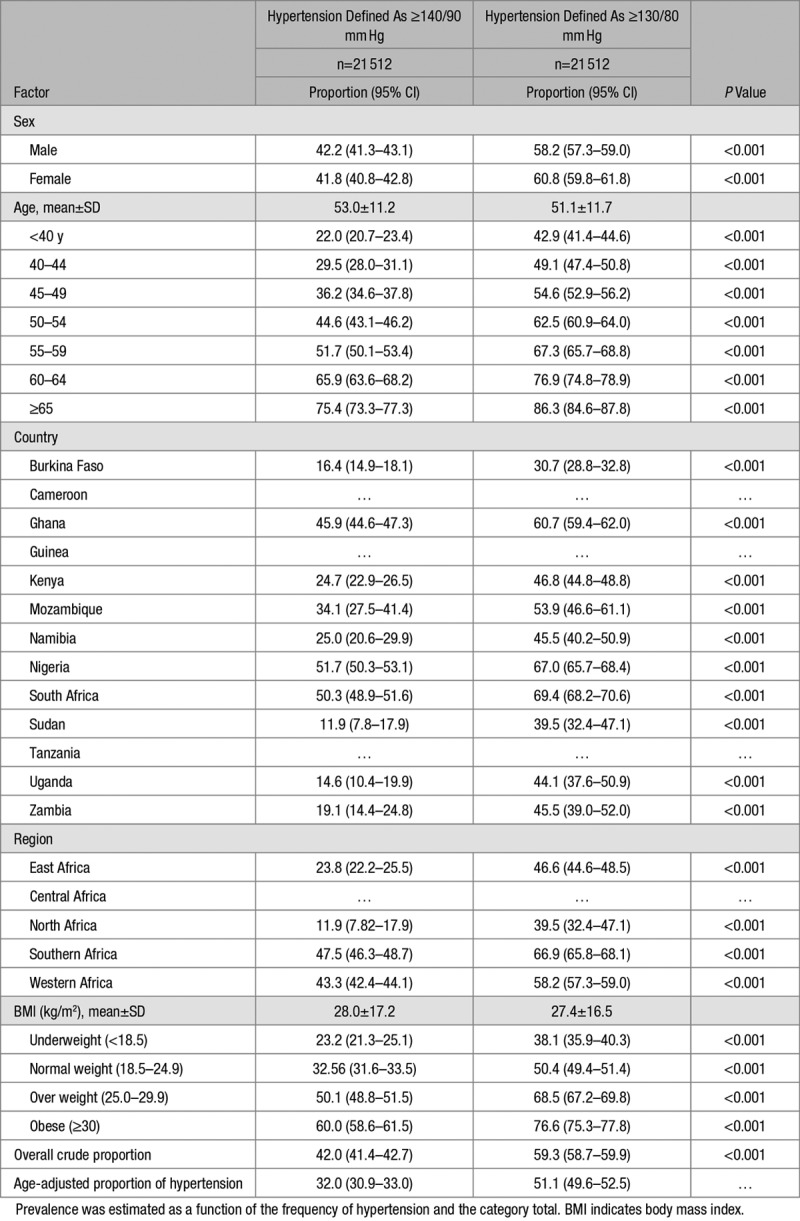
Crude and Age-Adjusted Proportion of Hypertension Stratified by Hypertension Definitions Per Sex, Age Group, Country Geographic Region, BMI, and Obesity in the CHAIR Population Controls (PC)

**Table 3.**
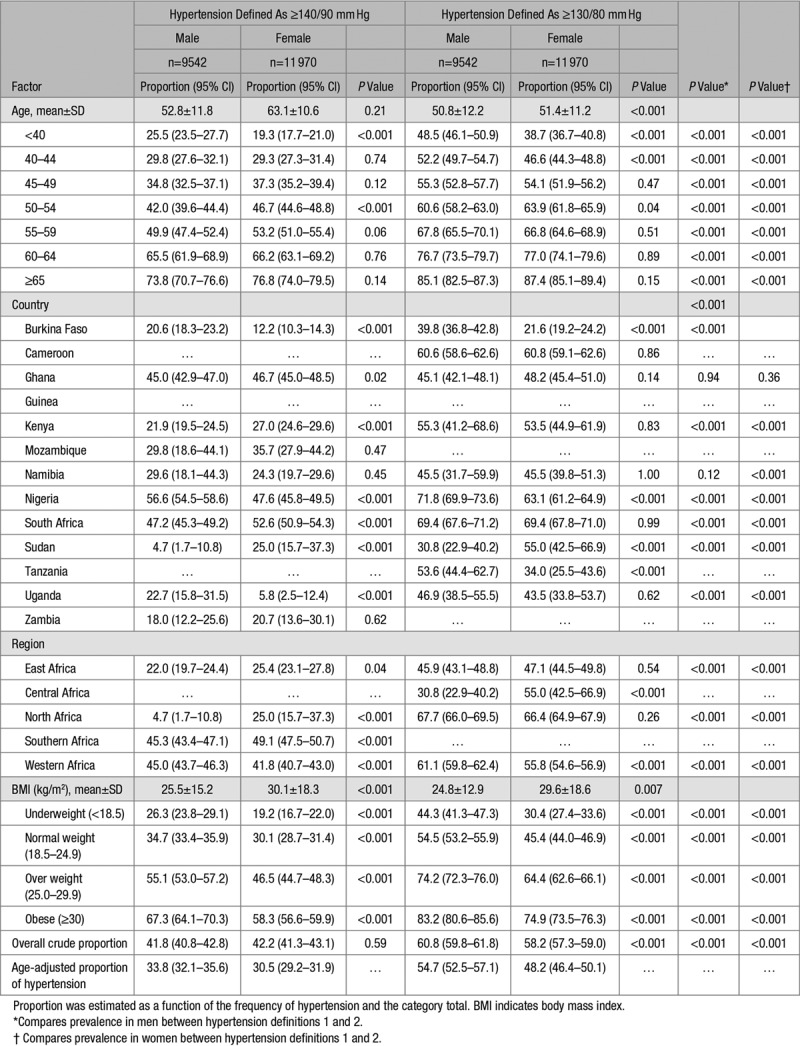
Proportion of Hypertension Among Population Controls (PC) Stratified by Sex Per Age Group, Country, Geographic Region, BMI, and Obesity

### Regional Differences in the Burden of Obesity and Hypertension

**Figure 1.**
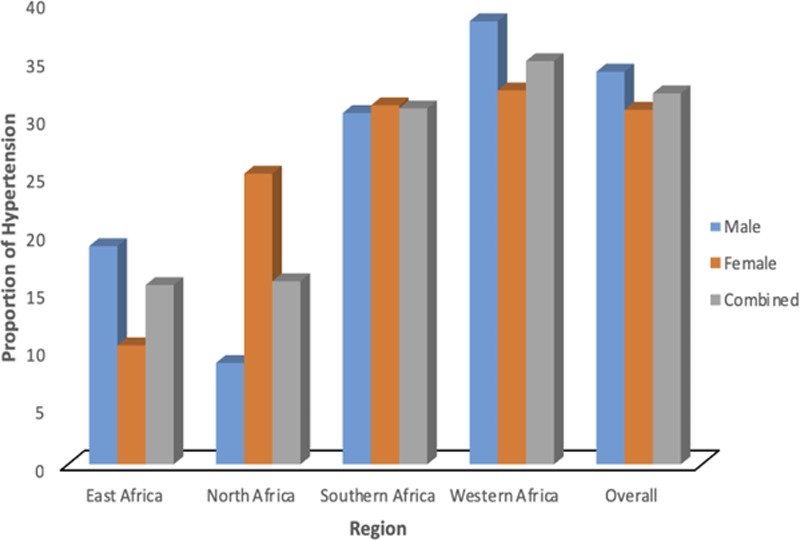
Regional differences in the age-adjusted proportion of hypertension using only the population controls dataset (PC) (for male, female, and the combined samples).

The PC data showed the regional age-adjusted proportion of hypertension as being marginally higher in western Africa (Figure 1) than the overall group and all other African regions included in the analysis. Hypertension rates were also the highest among older participants across regions but were relatively similar between men and women across age categories within regions (Figures S5 and S6). Similarly, the age-adjusted proportion of obesity in both sexes was higher in southern Africa than any other African region included in the analysis; with estimates being significantly higher in women than men (Figure S5). There were differences between countries in the same region (Figure S2). The proportion of hypertension was also the highest in PC participants with higher BMI across regions but were relatively similar between men and women within regions (Figure 4 [≥140/90 mm Hg] and Figure 5 (≥130/80 mm Hg)].

Regionally, the proportion of hypertension in both the EHD (Table S2) and the PC (Table 2) was consistently and substantially higher using the new 2017 definition. In the Eastern region for instance, the proportion of hypertension (57.4% [55.9–58.9]) using the new definition of hypertension was substantially higher than the estimate obtained using the old definition (36.1% [34.6–37.6]) for the EHD. Likewise, in PC, the proportion of hypertension was significantly higher for the new definition (66.9% [65.8–68.1]) compared with the old (47.5% [46.3–48.7]) in the Southern region of Africa.

Bivariate analysis revealed sex-dependent regional differences in hypertension and obesity: the proportion with hypertension was significantly higher in women than men in eastern Africa (25.4% [ 23.1–27.8] versus 22.0% [19.7–24.4]) and southern Africa (49.1% [47.1–50.7] versus 45.3% [43.4–47.1]) but higher among men than women in western Africa (45.0% [43.7–46.3] versus 41.8% [40.7–43.0]; Table 3). The proportion with obesity was also significantly higher in women than men in all regions of Africa and overall (Figure S1).

### Association Among Obesity, Hypertension, and Sex Across Age Groups

The sex-stratified proportions of hypertension showed significantly higher results among obese men compared with women in the EHD (72.4% [69.9–74.7] for men and 62.5% [61.2–63.8] for women; Table S3) as well as in the PC (67.3% [64.1–70.3] for men and 58.2% [56.6–59.9] for women; Table 3). The aOR of hypertension in the presence of obesity was consistently higher among women [from 2.1 (1.8–2.4) to 14.1 (12.1–16.5)] than men [from 2.0 (1.7–2.3) to 8.8 (7.4–10.3)], across all age categories (Figure S7; Table S4).

**Figure 2.**
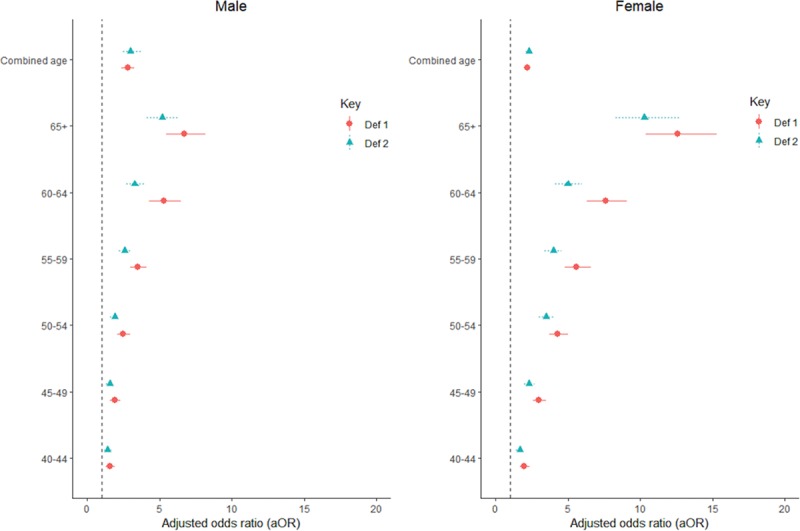
Adjusted odds of hypertension across different age categories and in obesity (body mass index >30; defined by ≥140/90 mm Hg [htn def1] and ≥130/80 mm Hg [htn def2]) stratified by the sex of participant. Age group <40 y was the reference age group in the analysis. def1 indicates hypertension defined as ≥140/90 mm Hg; and def2, hypertension defined as ≥130/80 mm Hg.

Specifically, in both sexes, the aOR of hypertension in obesity was consistently higher in older age groups (compared with younger participants—aged <40 years), and ranged from 2.0 (1.7–2.3) among younger men aged 40 to 44 years to 8.8 (7.4–10.3) among older men, aged ≥65 years in the EHD (Figure S3; Table S4). Similarly, compared with younger women (aged <40 years), aOR of hypertension in obesity ranged from 2.1 (1.8 to 2.4) among participants aged 40 to 44 years to as high as 14.1 (12.1 to 16.5) among older participants, aged ≥65 years (Figure 2; Table S3). Similar results were obtained for men and women participants in the PC (Figure 2; Table 4).

**Table 4.**
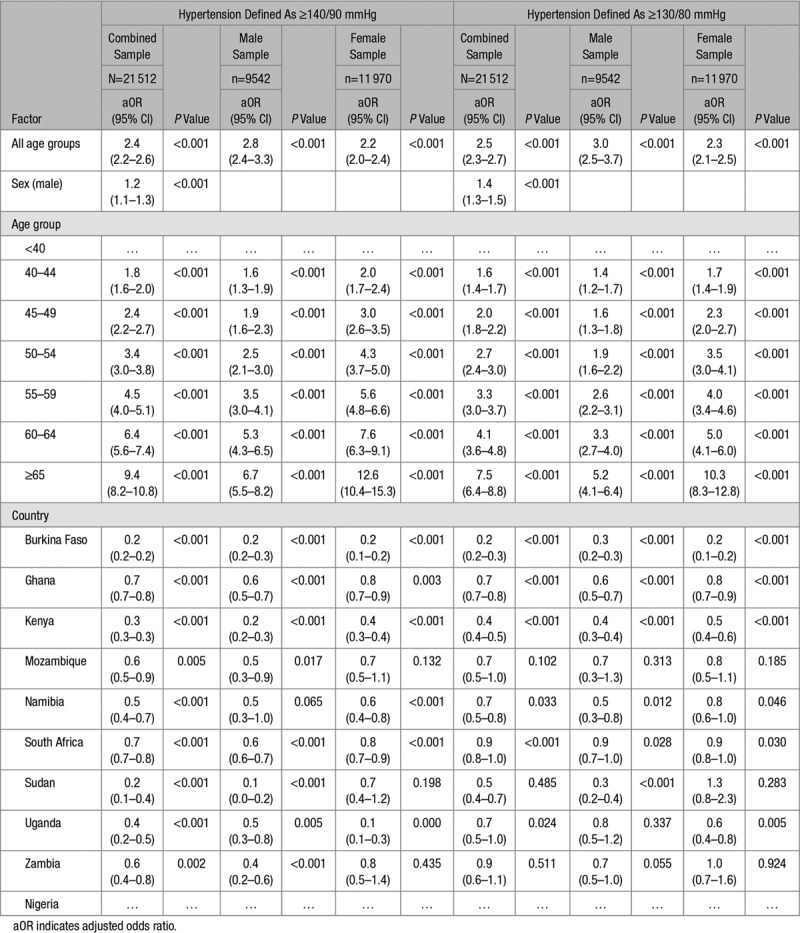
Association Between Obesity and Hypertension Adjusted for Sex, Age, and Country of Residence in the Population Control

Overall, obese participants were twice more likely to be hypertensive in the EHD (aOR, 2.4 [95% CI, 2.2–2.5]; Table S1) and the PC (aOR, 2.4 [95% CI, 2.2–2.6]; Table 4). Compared with Nigeria, the odds of hypertension in obese participants was 30% less in Ghana and South Africa but 80% less in Burkina Faso and Sudan (Table 4). Obese participants who were aged 65 years and above were 9× more likely to be hypertensive compared with their counterparts who were aged 40 years or less (Table 4).

## Discussion

Hypertension and obesity are extremely common across the 13 African countries in our study, with the highest age-adjusted proportion in Western Africa and among men. Obesity was significantly associated with a >2-fold increase in hypertension among both men and women, and across Africa. The large sample size and the high proportion of hypertension and obesity makes the case for policy makers to design interventions to address these 2 important cardiovascular risk factors in Africa.

### Burden and Regional Pattern of Hypertension and Obesity

Hypertension prevalence is diverse across African populations, ranging from 37% to 75%.^19,20^ We extend these findings to demonstrate that, overall, in an age-adjusted analysis, men had significantly higher proportions of hypertension compared with women, but there was some regional variation across Africa. Across WHO regions, the prevalence of hypertension has been reported to be the highest in Africa, 46% for both sexes combined for those over 25 years old.^21^ Also, while a study covering 4 countries in Africa reported an overall crude prevalence of 36.8%^19^ for Africa in 2015, it has been suggested that the prevalence of hypertension is increasing rapidly in Africa and shows regional differences similar to findings in our analyses.^22^ In South Africa for instance, among individuals aged 40 to 60 years in urban Soweto, crude prevalence of hypertension was found to be 54%^18^ while a recent systematic review revealed that hypertension prevalence was 51.6% in urban and 43% in rural areas in Nigeria.^23^ Differences in reported hypertension prevalence across regions may indicate that prevalence is unstable; might not have been accurately estimated due to limitations of power and coverage; or varies according to rural, urban, and regional differences. Thus, the proportions of hypertension from the population-based controls that exclude specific diseases in the present study are expected to be more reflective of the prevalence of hypertension in the communities from which the participants were recruited.

We observed that although population-based crude proportion of hypertension was the highest in southern Africa, the age-adjusted proportion of hypertension was the highest in Western Africa and among older participants across all regions. Hypertension prevalence has been reported to differ with geographic variations across countries in Africa. A previous review^24^ had reported disparities in (crude) hypertension prevalence for selected African countries and settings: rural Nigeria (14.5%), urban Eritrea (16.5%), urban Cameroon (19.1%), South Africa (26.9%), Ghana (29.4%), rural Tanzania (31.5%), and the Gambia (18.4%).

Furthermore, the age-adjusted proportion of obesity was the highest for women in Mozambique and South Africa and regionally higher for both sexes in southern Africa than any other African region covered in the population controls (PC) dataset with rates generally being higher among women than men. Previous reports corroborate our finding that the proportion of obesity was the highest in southern Africa.^25^ We hypothesized in this context that the regional differences in both obesity and hypertension may be attributable to disparities in socioeconomic, lifestyle, nutritional, and environmental risk factors at the regional level as well as genetic factors. These factors will be explored further in CHAIR.

### Association Between Obesity and Hypertension Across Africa

We found that across all age groups, obese individuals were at least twice as likely to be hypertensive compared with nonobese participants and the situation was worse in South Africa compared with any other country. Overall, the adjusted odds of hypertension in obesity increased with increasing age in both sexes. We did not find any previous report describing such trend in all publications reviewed for this work.

Previous smaller studies have reported variable associations between BP and BMI but have not demonstrated the impact of age on the association between BP and obesity. For instance, an increase of 1.71 mm Hg per 1 kg/m^2^ for systolic BP was reported in West Africa with the increase being higher in men than women,^26^ while a BMI greater than 25 kg/m^2^ increased the odds of hypertension by 50% in urban Nigeria.^2^ As a result of the large sample size and geographic spread of the present analysis, we infer that a more accurate and stronger association was found between obesity and hypertension across different age strata, sex, regions and comorbid disease conditions in Africa. The association was stronger in women and with increasing age. Proposed pathways underlying this association include activation of the rennin-angiotensin-aldosterone system and increased procoagulatory activity, insulin resistance, sympathetic activity, leptin resistance, and endothelial dysfunction.^27^

Strategies for managing obesity-related hypertension on the continents has been poor with most aimed mainly at treating the hypertension and other metabolic consequences of obesity, such as dyslipidemia and diabetes mellitus.^28^ Interventions specifically targeting obesity-related hypertension are needed on the continent and such intervention strategies should aim to treat the underlying causes of obesity.^29^ Primary prevention targeting the development of effective, culturally sensitive strategies for the prevention and treatment of obesity will benefit patients in Africa. In particular, promoting an obesity-preventative lifestyle and behavioral educational interventions emphasizing quality diet, increased physical activity, reduced alcohol consumption and other behavior modifications have been suggested by Kotchen. In addition to genetic determinants of obesity-related hypertension, other ways of addressing this condition in Africa includes implementation of clinical trials to identify more appropriate drug therapies for reducing obesity-induced hypertension.^29^

### Impact of the 2017 Definition of Hypertension

Using the 2017 definition, the burden of hypertension was unsurprisingly substantially higher, with doubling of the population burden in some groups, in both the EHD and PC. The new guideline generated discussions in the field of hypertension research worldwide.^12,30,31^ For instance, using these criteria, nearly half (crude prevalence of 46%) of American adults (aged>18 years) have hypertension (compared with 32% based on previous guidelines)^30^ and 63% of individuals in the 45- to 75-year age group are considered hypertensive.^31^ By implication, government spending on prevention and treatment of hypertension and associated health problems will increase astronomically. What is important, however, is to understand the 10-year risk prediction for CVD end points given the new cut-points for values and whether they would affect risk for CVD in a similar way among Africans. Judging from the results of the present study, the 2017 guidelines for hypertension diagnosis has important implications for the treatment and management of hypertension in Africa as the estimated crude proportion of hypertension (64.0%) in Africa poses a huge challenge. In particular, this result shows an urgent need for concerted population-based health education to address important risk factors such as diet and the need for reduced salt intake as measures for primary and primordial prevention of hypertension. Previous reports from Africa have shown that reducing the sodium content of food has potential for positive population–based public health effects.^32^

### Strengths, Limitations, and Future Directions

Our study has some limitations. The disparities in study design across the 5 studies reflect their specific individual recruitment strategies and we have attempted to mitigate differences in data collection by performing rigorous data harmonization. In addition, we analyzed the data in 2 ways: for the EHD and then only for the disease-free and community-based PC individuals. The latter was an attempt to reduce disease-related bias. The inclusion of cases with stroke, chronic kidney disease, and diabetes mellitus in the EHD have led to the observed higher proportion of hypertension and obesity in the EHD compared with the PC group. In addition, the proportion of hypertension and obesity in the PC group may only partially reflect the true population prevalence of hypertension and obesity in the respective communities because none of the studies recruited population-based participants using a door-to-door survey. Also, most of the data used in the present study were derived from tertiary health institutions located in urban areas, there is the likelihood that the data will be skewed toward urban dwellers with likely higher prevalence of hypertension and obesity compared with if data were taken from the general population. Nevertheless, consistently, an almost identical relationship between hypertension and obesity was observed in the EHD and the PC.

Furthermore, we defined obesity as BMI≥30 kg/m^2^ in the present study, but where data are available, we plan to examine central obesity by using the waist circumference ratio in future because BMI is a suitable index in evaluating overall weight changes but weak in discerning endogenous alterations of fat localization. Also, the 2017 revised definition of hypertension for CVD risk was based on data from outside the African continent and may not equally apply in Africa. BP is a continuous trait and various definitions/thresholds for disease risk are used at different times, which even though evidence-based, are artificial thresholds. We herein explored the implication of applying the lower threshold for countries in Africa.

Our study, which is the largest empirical study on hypertension and obesity in Africa thus far, covering 13 countries and 30 044 participants, has contributed new knowledge on the regional patterns and burden of hypertension and obesity across African regions. We have quantified the association between hypertension and obesity across different subpopulations. We have also demonstrated the impact of the new hypertension definition in Africans. The regional and sex disparities observed will be investigated further using the CHAIR resource to unravel the contributions of genetic and epigenetic factors, as well as emerging environmental and lifestyle factors such as dietary, psychosocial and economic factors, and environmental pollution.

### Perspectives

We have shown that obesity plays an important role in the sex and regional differences observed in the proportions of hypertension. There was a stronger association between obesity and hypertension in women compared with men. In conclusion, hypertension is common across the African continent with >30% having age-adjusted BP≥140/90 mmHg and >50% with age-adjusted BP≥130/80 mmHg. Proportions with hypertension differ significantly by region, country, age, and sex. In particular, Southern Africa had the highest crude proportion (and Western Africa had the highest age-adjusted proportion) of hypertension and hypertension was more common among obese participants and men. Obesity is associated with at least twice the risk for hypertension among Africans; this risk increases with age and is higher in women. It is anticipated that interventions addressing obesity will likely lead to more effective control of hypertension in Africa.

## Acknowledgments

We acknowledge all participants, site research assistants, and other scientists who participated in the data collection for the studies. We also acknowledge Dr Jean-Tristan Brandenburg for assisting with the forest plots. Co-authors contributed to one or more of the following: study concept, design, and/or data acquisition. O.M. Akpa and FM are Cardiovascular H3Africa Innovation Resource (CHAIR) statisticians who contributed to data harmonization and analysis. M.O. Owolabi, M. Ramsay, O.M. Akpa, and FM contributed to interpretation of results and drafting of the manuscript. All authors contributed to critical revision of the manuscript and approval of the final draft. Additional information: Coauthor Bongani M. Mayosi, MBChB, DPhil contributed to data collection, reading and approving of the draft manuscript before his death.

## Sources of Funding

The harmonization process for the Cardiovascular H3Africa Innovation Resource consortium is supported by the administrative National Institutes of Health (NIH) supplement U54HG007479-03S1 to the SIREN study for the benefit of the H3Africa CVD Working Group. SIREN is funded by the NIH (Grant U54HG007479; NINDS/NHGRI/NIEHS); Systematic Investigation of Blacks with Stroke using Genomics (SIBS Genomics) NIH (NINDS/NHGRI), R01NS107900; African Neurobiobank for Precision Stroke Medicine—(ELSI) Project NIH 1U01HG010273; Africa-UK Collaboration for the genetic Epidemiology of Stroke (ACES) is funded by the Academy of Medical Sciences Global Challenges Research Fund Networking Grant Scheme (GCRFNGR2\10190). The AWI-Gen Collaborative Centre is funded by the NIH (NHGRI; NICHD; OD; Grant U54HG006938). The ACCME cohort is funded by the NIH (NHGRI grant U54HG006947). The H3Africa Kidney Disease Research Consortium if funded by the NIH (Grant U54HG006939). The DM Study (Burden, spectrum, and etiology of type 2 diabetes mellitus in sub-Saharan Africa) is funded by the Wellcome Trust (Grant No. WT 099316AIA). The RHDGen Network is funded by the Wellcome Trust (099313/Z/12/Z and 099313/B/12/Z). C. Rotimi is supported by the intramural program of the NHGRI/NIH at the Center for Research on Genomics and Global Health (CRGGH); The CRGGH is also supported by NIDDK, CIT, and the NIH Office of the Director. R.S. Parekh is funded by the Canada Research Chair in Chronic Kidney Disease Epidemiology. The content is solely the responsibility of the authors and does not necessarily represent the official views of the National Institutes of Health and the Wellcome Trust. The funders played no role in the data collection or interpretation of this study.

## Disclosures

None.

## Supplementary Material



## Novelty and Significance

### What Is New?

- This is the largest and the first study to investigate the association between hypertension and obesity across 13 African countries in a single report.
- The burden of hypertension in Africa was substantially higher using the new 2017 definition compared with the previous definition for hypertension.
- Regionally, the crude proportion of hypertension was the highest in southern Africa while age-adjusted proportion of hypertension was the highest in Western Africa.
- There were sex-dependent regional differences in the burden of hypertension and obesity: the proportion with hypertension was significantly higher among women than men in eastern Africa, northern Africa, and southern Africa.
- Obese participants were consistently twice as likely to be hypertensive (adjusted odds ratio, 2.5 [2.3–2.7]) compared with nonobese participants and the risk increases with increasing age in both sex.

### What Is Relevant?

- The burden of hypertension in Africa is particularly high compared with other world regions. Previous smaller studies have found associations between hypertension and obesity in selected countries in sub-Saharan Africa.
- However, this relationship has not been examined rigorously and simultaneously across African countries.
- Moreover, the extent to which the new lower threshold for diagnosing hypertension will affect the estimates of the burden of hypertension and its pattern and association with obesity in African populations is unknown.

### Summary

- The proportion of crude and age-adjusted proportion of hypertension in Africa is high, substantially higher with the 120/80 mmHg definition, with regional differences and in some cases sex-specific differences.
- Obesity doubles the risk of hypertension in Africa and the risk increases with increasing age in both sex.
- Interventions targeting obesity may help to significantly reduce the overwhelming burden of hypertension on the continent.
