# Supplementary material for: Regional Patterns and Association Between Obesity and Hypertension in Africa: Evidence From the H3Africa CHAIR Study
Source: Hypertension. 2020 Mar 16;75(5):1167–78. doi: 10.1161/HYPERTENSIONAHA.119.14147 (PMC7176339; doi:10.1161/HYPERTENSIONAHA.119.14147)
Supplement: Supplementary file 1 [file hyp-75-1167-s001.doc]

**Regional patterns and association between obesity and hypertension in Africa: 30,044 participants from the H3Africa CHAIR study**

**Authors and affiliations:**

Onoja M. Akpa, Ph.D.^1-3,5^, Felix Made, M.Sc.^4,5^, Akinlolu Ojo, MD, Ph.D.^6^, Bruce Ovbiagele MD, FAAN^7^, Dwomoa Adu MD^8^, Ayesha A. Motala, MD^9^, Bongani M. Mayosi, MBChB, DPhil^10&^, Sally N. Adebamowo, MD, ScD^11^, Mark E. Engel, MPH, Ph.D.^12^, Bamidele Tayo, PhD^13^, Charles Rotimi, Ph.D.^14^, Babatunde Salako, MD^1^ Rufus Akinyemi MBBS, Ph.D.^1^, Mulugeta Gebregziabher, Ph.D.^15^, Fred Sarfo MBBS, Ph.D.^16^, Kolawole Wahab MBBS, MPH^17^, Godfred Agongo, MPhil^18^, Marianne Alberts, Ph.D.^19^, Stuart A Ali, Ph.D.^5^, Gershim Asiki, Ph.D.^20^, Romuald P Boua, M.Sc.^21^, F Xavier Gómez-Olivé, Ph.D.^22^, Felistas Mashinya, Ph.D.^19^, Lisa Micklesfield, Ph.D.^23^, Shukri F Mohamed, PharmD, MPH^20^, Engelbert A Nonterah, MBchB,M.Sc.^18^, Shane A Norris, Ph.D.^23^, Hermann Sorgho, Ph.D.^21^, Stephen Tollman, MPH, Ph.D.^22^, Rulan S. Parekh, MD,MS^24^, Chisala Chisala^12^, Kenneth Ekuro, Ph.D.^25^, Salina P. Waddy, MD^26^, Emmanuel Peprah Ph.D.^27^, George A. Mensah MD^28^, Ken Wiley Ph.D.^29^, Jennifer Troyer Ph.D^30^, Michèle Ramsay, Ph.D.^5#^ Mayowa O. Owolabi MBBS, MSc, DrM, FAAN, FAS^1*#^,as members of the CVD Working Group of the H3Africa Consortium

^&^ Deceased

*^#^ Joint senior authors*

**Affiliations:**

1. Center for Genomic and Precision Medicine, University of Ibadan (O.M.A., R.A., B.S., M.O.O.)
2. Department of Epidemiology and Medical Statistics, College of Medicine, University of Ibadan (O.M.A.)
3. Institute of Cardiovascular Diseases, College of Medicine, University of Ibadan (O.M.A.)
4. The Epidemiology and Surveillance Section, National Institute for Occupational Health, National Health Laboratory Services, Gauteng Region, South Africa (F.M.)
5. Sydney Brenner Institute for Molecular Bioscience and Division of Human Genetics, Faculty of Health Sciences, University of the Witwatersrand, Johannesburg, South Africa (O.M.A. F.M., S.A.A., M.R.)
6. Clinical research and global health initiatives, University of Arizona Health Sciences (A.O.)
7. Department of Neurology, University of California, San Francisco (B.O.)
8. School of Medicine and Dentistry, University of Ghana. P.O. Box 4236, Accra, Ghana (D.A.)
9. Department of Diabetes and Endocrinology, Nelson R. Mandela School of Medicine, University of KwaZulu-Natal, Durban, South Africa (A.A.M.)
10. Department of Medicine, Groote Schuur Hospital and University of Cape Town, Cape Town, South Africa (B.M.M.)
11. Department of Epidemiology and Public Health; and Greenebaum Comprehensive Cancer Center, University of Maryland School of Medicine, Baltimore, MD 21201 (S.N.A)
12. Division of Cardiology, Department of Medicine, University of Cape Town, Cape Town, South Africa (M.E.E., C.C.)
13. Department of Preventive Medicine and Epidemiology, Loyola University Chicago Stritch School of Medicine, Maywood, Illinois, United States of America (B.T.)

14. Center for Research on Genomics and Global Health, NHGRI, NIH, Bethesda, Maryland, USA (C.R.)

15. Department of Public Health Sciences, Medical University of South Carolina, Charleston, USA (M.G.)

16. Kwame Nkrumah University of Science and Technology, Kumasi, Ghana (F.S.)

17. Department of Medicine, University of Ilorin, Ilorin, Nigeria (K.W.)

18. Navrongo Health Research Centre, Navrongo, Ghana (G.A., E.A.N.)

19. Department of Pathology and Medical Science, School of Health Care Sciences, Faculty of Health Sciences, University of Limpopo, Polokwane, South Africa (M.A., F.M.)

20. African Population and Health Research Center, Nairobi, Kenya (G.A. S.F.M.)

21. Institut de Recherche en Sciences de la Sante, Clinical Research Unit of Nanoro, Burkina Faso (R.P.B., H.S.)

22. MRC/Wits Rural Public Health and Health Transitions Research Unit (Agincourt), School of Public Health, Faculty of Health Sciences, University of the Witwatersrand, Johannesburg 2193, South Africa (F.G., S.T.)

23. MRC/Wits Developmental Pathways for Health Research Unit, Faculty of Health Sciences, University of the Witwatersrand, Johannesburg, South Africa (L.M., S.A.N.)

24. Departments of Pediatrics, Medicine and Epidemiology, Hospital for Sick Children, University Health Network and University of Toronto (R.S.P.)

25. Center for Research on Genomics and Global Health, National Human Genome Research Institute, National Institutes of Health (K.E.)

26. Department of Neurology, Atlanta Veterans Affairs Medical Center, Decatur, Georgia, USA

27. New York University, College of Global Public Health, U.S.A. (E.P.)

28. Center for Translation Research and Implementation Science, National Heart, Lung, and Blood Institute, NIH, Bethesda, USA (G.A.M.)

29. Division of Genomic Medicine, National Human Genome Research Institute, National Institutes of Health, USA (K.W.)

30. Human Heredity and Health in Africa, Division of Genome Sciences, National Institutes of Health, USA (J.T.)

*Correspondence

**Prof. Mayowa O. Owolabi, *MBBS, MSc, DrM, FAAN, FANA, FRCP, FAS***

Department of Medicine and Center for Genomic and Precision Medicine,

University of Ibadan, Ibadan, Nigeria

[mayowaowolabi@yahoo.com](mailto:mayowaowolabi@yahoo.com)

**Supplemental Materials**

**Table S1. Demographic, obesity and hypertension information stratified by sex and assessing significant sex-related differences in the CHAIR Entire Harmonized Dataset (EHD)**

| **Factors** | **Total**  **N=30044** | **Male**  **n=12921** | **Female**  **n=17123** | **P*** |  |
| --- | --- | --- | --- | --- | --- |
| **Sex** |  |  |  |  |  |
| *Male* | 12921(43.0) |  |  |  |  |
| *Female* | 17123(57.0) |  |  |  |  |
| ***Total*** | **30044** |  |  |  |  |
| **Age, mean ± SD** | **48.9 ±13.2** | **49.2±13.4** | **48.8±13.0** | **0.008** |  |
| *<40* | 6036(20.1) | 2518 (41.7) | 3518 (58.3) | <0.001 |  |
| *40-44* | 4223(14.1) | 1823 (43.2) | 2400 (56.8) | <0.001 |  |
| *45-49* | 4515(15.0) | 1922 (42.6) | 2593 (57.4) | <0.001 |  |
| *50-54* | 4829(16.1) | 2041 (42.3) | 2788 (57.7) | <0.001 |  |
| *55-59* | 4535(15.1) | 1952 (43.0) | 2583 (57.0) | <0.001 |  |
| *60-64* | 2558(8.5) | 1100 (43.0) | 1458 (57.0) | <0.001 |  |
| *≥65* | 3348(11.1) | 1565 (46.7) | 1783 (53.3) | <0.001 |  |
| ***Total*** | **30044** | **12921** | **17123** |  |  |
| **Country (%)** |  |  |  |  |  |
| *Burkina Faso* | 2082(6.9) | 1049 (50.4) | 1033 (49.6) | 0.620 |  |
| *Cameroon* | 117(0.4) | 41 (35.0) | 76 (65.0) | <0.001 |  |
| *Ghana* | 6666(22.2) | 2942 (44.1) | 3724 (55.9) | <0.001 |  |
| *Guinea* | 74(0.2) | 21 (28.4) | 53 (71.6) | <0.001 |  |
| *Kenya* | 2555(8.5) | 1119 (43.8) | 1436 (56.2) | <0.001 |  |
| *Mozambique* | 358(1.2) | 98 (27.4) | 260 (72.6) | <0.001 |  |
| *Namibia* | 481(1.6) | 83 (17.3) | 398 (82.7) | <0.001 |  |
| *Nigeria* | 7826(26.0) | 3711 (47.4) | 4115 (52.6) | <0.001 |  |
| *South Africa* | 7880(26.2) | 3033 (38.5) | 4847 (61.5) | <0.001 |  |
| *Sudan* | 340(1.1) | 158 (46.5) | 182 (53.5) | 0.070 |  |
| *Tanzania* | 572(1.9) | 221 (38.6) | 351 (61.4) | <0.001 |  |
| *Uganda* | 780(2.6) | 295 (37.8) | 485 (62.2) | <0.001 |  |
| *Zambia* | 313(1.0) | 150 (47.9) | 163 (52.1) | 0.300 |  |
| ***Total*** | **30044** | **12921** | **17123** |  |  |
| **Region** |  |  |  |  |  |
| *East Africa* | 3907(13.0) | 1635 (41.9) | 2272 (58.2) | <0.001 |  |
| *Central Africa* | 117(0.4) | 41 (35.0) | 76 (65.0) | <0.001 |  |
| *North Africa* | 340(1.1) | 158 (46.5) | 182 (53.5) | 0.070 |  |
| *Southern Africa* | 9032(30.1) | 3364 (37.3) | 5668 (62.8) | <0.001 |  |
| *Western Africa* | 16648(55.4) | 7723 (46.4) | 8925 (53.6) | <0.001 |  |
| ***Total*** | **30044** | **12921** | **17123** |  |  |
| **BMI (kg/m^2^), mean±SD** | **26.6 ±16.3** | **24.5±16.7** | **28.2±15.8** | **<0.001** |  |
| *Underweight (<18.5)* | 2275(7.9) | 1229 (54.0) | 1046 (46.0) | <0.001 |  |
| *Normal weight (18.5-24.9)* | 12519(43.4) | 6718 (53.7) | 5801 (46.3) | <0.001 |  |
| *Over weight (25.0-29.9)* | 7325(25.4) | 3077 (42.0) | 4248 (58.0) | <0.001 |  |
| *Obese (≥30)* | 6732(23.3) | 1332 (19.8) | 5400 (80.2) | <0.001 |  |
| **Total** | **28851^ƺ^** | **12356** | **16495** |  |  |
| **Proportion of Hypertension defined as** *≥***140/90** | 14401(48.0) | 6297 (21.0) | 8104 (27.0) | <0.001 |  |
| **Proportion of Hypertension defined as** *≥***130/80** | 19196(64.0) | 8493(44.2) | 10703(55.8) | <0.001 |  |

***ƺ – Number do not add up due to missing observations***

*BMI – Body Mass Index*

** P - comparison between males and females*

**Table S2. Crude and age-adjusted proportion of hypertension stratified by hypertension definitions per sex, age group, country geographic region, BMI and obesity in the CHAIR Entire Harmonized Data (EHD)**

|  | **Hypertension defined as ≥140/90 mmHg** |  | **Hypertension defined as ≥130/80 mmHg** |  |  |  |
| --- | --- | --- | --- | --- | --- | --- |
|  | **CHAIR Combined Samples (EHD)** |  | **CHAIR Combined Samples (EHD)** |  |  |  |
|  | **N=30044** |  | **N=30044** |  |  |  |
| **Factor** | **Proportion (95%CI)** |  | **Proportion (95%CI)** |  | **P** |  |
| **Sex** |  |  |  |  |  |  |
| *Male* | 47.3(46.6 -48.1) |  | 62.5(61.8-63.2) |  | <0.001 |  |
| *Female* | 48.7(47.87-49.6) |  | 65.7(64.9-66.5) |  | <0.001 |  |
| **Age, mean±SD** | **54.0±12.0** |  | **51.9±12.6** |  |  |  |
| *<40 years* | 23.9(22.9-25.0) |  | 44.9(43.7-46.2) |  | <0.001 |  |
| *40-44* | 34.1(32.6-35.5) |  | 53.4(51.9-54.9) |  | <0.001 |  |
| *45-49* | 41.7(40.3-43.2) |  | 59.0(57.6-60.5) |  | <0.001 |  |
| *50-54* | 51.0(49.6-52.4) |  | 67.3(65.9-68.6) |  | <0.001 |  |
| *55-59* | 57.9(56.4-59.3) |  | 71.8(70.5-73.1) |  | <0.001 |  |
| *60-64* | 71.7(69.9-73.5) |  | 81.6(80.0-83.0) |  | <0.001 |  |
| *≥65* | 80.9(79.6-82.3) |  | 88.8(87.7-89.9) |  | <0.001 |  |
| **Country** |  |  |  |  |  |  |
| *Burkina Faso* | 16.4(14.9-18.1) |  | 30.7(28.8-32.8) |  | <0.001 |  |
| *Cameroon* | 55.6(46.5-64.2) |  | 66.7(57.7-74.6) |  | 0.08 |  |
| *Ghana* | 53.0(51.8-54.2) |  | 66.0(64.9-67.2) |  | <0.001 |  |
| *Guinea* | 64.9(53.5-74.8) |  | 78.4(67.6-86.3) |  | 0.07 |  |
| *Kenya* | 23.3(21.7-25.0) |  | 46.9(45.0-48.9) |  | <0.001 |  |
| *Mozambique* | 24.9(20.7-29.6) |  | 46.1(40.9-51.3) |  | <0.001 |  |
| *Namibia* | 23.5(19.9-27.5) |  | 45.7(41.3-50.2) |  | <0.001 |  |
| *Nigeria* | 58.9(57.8-60.0) |  | 72.9(71.9-73.9) |  | <0.001 |  |
| *South Africa* | 52.1(51.0-53.3) |  | 69.2(68.2-70.2) |  | <0.001 |  |
| *Sudan* | 8.2(5.7-11.7) |  | 31.8(27.0-36.9) |  | <0.001 |  |
| *Tanzania* | 70.5(66.6-74.1) |  | 83.2(79.9-86.1) |  | <0.001 |  |
| *Uganda* | 52.7(49.2-56.2) |  | 72.8(69.6-75.8) |  | <0.001 |  |
| *Zambia* | 15.9(12.3-20.5) |  | 38.9(33.7-44.5) |  | <0.001 |  |
| **Region** |  |  |  |  |  |  |
| *East Africa* | 36.1(34.6-37.6) |  | 57.4(55.9-58.9) |  | <0.001 |  |
| *Central Africa* | 55.6(46.5-64.2) |  | 66.7(57.7-74.6) |  | 0.08 |  |
| *North Africa* | 8.24(5.7-11.7) |  | 31.8(27.0-36.9) |  | <0.001 |  |
| *Southern Africa* | 48.3(47.3-49.3) |  | 65.9(64.9-66.9) |  | <0.001 |  |
| *Western Africa* | 51.3(50.5-52.0) |  | 64.9(64.2-65.6) |  | <0.001 |  |
| **BMI (kg/m^2^), mean±SD** | **28.3±14.9** |  | **27.8±15.2** |  |  |  |
| *Underweight (<18.5)* | 24.6(22.9-26.4) |  | 39.9(37.9-41.9) |  | <0.001 |  |
| *Normal weight (18.5-24.9)* | 36.9(36.0-37.7) |  | 54.3(53.4-55.1) |  | <0.001 |  |
| *Over weight (25.0-29.9)* | 55.2(54.0-56.3) |  | 71.6(70.5-72.6) |  | <0.001 |  |
| *Obese (≥30)* | 64.5(63.365.6) |  | 79.1(78.2-80.1) |  | <0.001 |  |
| Overall crude proportion | 47.9(47.4-48.5) |  | 63.9(63.4-64.4) |  | <0.001 |  |
| **Age-adjusted proportion of hypertension** | **35.1(34.3-36.0)** |  | **53.7(52.6-54.9)** |  | **-** |  |

*Note - Prevalence was estimated as a function of the frequency of hypertension and the category total*

*BMI- Body mass index*

Table S3. Proportion of hypertension in the Entire Harmonized Dataset (EHD) and stratified by sex per age group, country geographic region, BMI and obesity

|  | **Hypertension defined as ≥140/90 mmHg** | | |  | **Hypertension defined as ≥130/80 mmHg** | | |  |  |  |  |
| --- | --- | --- | --- | --- | --- | --- | --- | --- | --- | --- | --- |
|  | **Male** | **Female** |  |  | **Male** | **Female** |  |  |  |  |  |
|  | **n=12921** | **n=17123** |  |  | **n=12921** | **n=17123** |  |  |  |  |  |
| **Factor** | **Proportion**  **(95%CI)** | **Proportion (95%CI)** | **P** |  | **Proportion (95%CI)** | **Proportion (95%CI)** | **P** |  | **P^1^** |  | **P^2^** |
| **Age (years)** | **53.8±12.4** | **54.1±11.7** | **0.17** |  | **51.7±13.0** | **52.1±12.3** | **0.05** |  |  |  |  |
| *<40* | 28.0(26.3-29.8) | 21.0(19.7-22.4) | <0.001 |  | 50.2(48.3-52.2) | 41.1(39.5-2 42.7) | <0.001 |  | <0.001 |  | <0.001 |
| *40-44* | 35.8(33.7-38.1) | 32.7(30.9-34.6) | 0.03 |  | 57.1(54.8-59.3) | 50.6(48.6-52.6) | <0.001 |  | <0.001 |  | <0.001 |
| *45-49* | 40.6(38.5-42.9) | 42.5(40.6-44.4) | 0.21 |  | 59.6(57.4-61.8) | 58.6(56.7-60.5) | 0.096 |  | <0.001 |  | <0.001 |
| *50-54* | 49.6(47.5-51.8) | 52.0(50.2-53.9) | 0.10 |  | 66.6(64.5-68.6) | 67.7(66.0-69.4) | 0.875 |  | <0.001 |  | <0.001 |
| *55-59* | 56.1(53.8-58.2) | 59.3(57.4-61.2) | 0.03 |  | 71.9(69.8-73.8) | 71.7(69.9-73.4) | 0.099 |  | <0.001 |  | <0.001 |
| *60-64* | 72.1(69.4-74.7) | 71.5(69.1-73.7) | 0.73 |  | 81.8(79.4-84.0) | 81.3(79.2-83.2) | 0.500 |  | <0.001 |  | <0.001 |
| *≥65* | 80.3(78.3-82.2) | 81.6(79.7-83.3) | 0.37 |  | 88.3(86.6-89.8) | 89.2(87.7-90.6) | 0.182 |  | <0.001 |  | <0.001 |
| **Country** |  |  |  |  |  |  |  |  |  |  |  |
| *Burkina Faso* | 20.6(18.3-23.2) | 12.2(10.3-14.3) | <0.001 |  | 39.8(36.8-42.8) | 21.5(19.1-24.2) | 0.156 |  | <0.001 |  | <0.001 |
| *Cameroon* | 58.5(43.4-72.3) | 54.0(42.8-64.7) | 0.63 |  | 78.1(63.1-88.2) | 60.5(49.2-70.7) | 0.000 |  | 0.06 |  | 0.41 |
| *Ghana* | 54.0(52.2-55.8) | 52.3(50.7-53.9) | 0.17 |  | 67.2(65.5-68.9) | 65.1(63.5-66.6) | 0.005 |  | <0.001 |  | <0.001 |
| *Guinea* | 47.6(28.3-67.6) | 71.7(58.4-82.1) | 0.05 |  | 61.9(40.8-79.3) | 84.9(72.6-92.4) | 0.125 |  | 0.35 |  | <0.001 |
| *Kenya* | 21.5(19.2-24.0) | 24.7(22.6-27.0) | 0.06 |  | 45.8(42.9-48.8) | 47.7(45.2-50.3) | 0.320 |  | <0.001 |  | <0.001 |
| *Mozambique* | 24.5(17.0-33.9) | 25.0(20.1-30.6) | 0.92 |  | 46.9(37.4-56.8) | 45.7(39.8-51.8) | 0.037 |  | <0.001 |  | <0.001 |
| *Namibia* | 30.1(21.3-40.7) | 22.1(18.3-26.5) | 0.12 |  | 54.2(43.6-64.5) | 43.9(39.1-48.8) | 0.000 |  | <0.001 |  | <0.001 |
| *Nigeria* | 64.4(62.9-65.9) | 54.0(52.5-55.5) | <0.001 |  | 77.5(76.1-78.8) | 68.8(67.4-70.2) | 0.147 |  | <0.001 |  | <0.001 |
| *South Africa* | 47.7(45.9-49.5) | 54.9(53.5-56.3) | <0.001 |  | 68.1(66.4-69.8) | 69.8(68.5-71.1) | 0.023 |  | <0.001 |  | <0.001 |
| *Sudan* | 5.1(2.4-9.8) | 11.0(7.2-16.4) | 0.05 |  | 29.1(22.6-36.6) | 34.0(27.5-41.2) | <0.001 |  | <0.001 |  | <0.001 |
| *Tanzania* | 66.5(60.1-72.4) | 72.9(68.1-77.3) | 0.10 |  | 81.5(75.8-86.1) | 84.3(80.1-87.7) | 0.893 |  | <0.001 |  | <0.001 |
| *Uganda* | 51.2(45.5-56.8) | 53.6(49.2-58.0) | 0.50 |  | 72.9(67.5-77.6) | 72.7(68.6-76.5) | 0.209 |  | <0.001 |  | <0.001 |
| *Zambia* | 18.0(12.6-25.0) | 14.1(9.5-20.4) | 0.30 |  | 46.0(38.2-54.0) | 32.5(25.7-40.0) | <0.001 |  | <0.001 |  | <0.001 |
| **Region** |  |  |  |  |  |  |  |  |  |  |  |
| *East Africa* | 33.0(30.7-35.3) | 38.3(36.4-40.4) | <0.001 |  | 55.5(53.1-57.9) | 58.7(56.7-60.7) | 0.432 |  | <0.001 |  | <0.001 |
| *Central Africa* | 58.5(43.4-72.3) | 54.0(42.8-64.7) | 0.60 |  | 78.1(63.1-88.2) | 60.5(49.2-70.7) | 0.156 |  | 0.06 |  | 0.41 |
| *North Africa* | 5.1(2.4-9.8) | 11.0(7.2-16.4) | 0.05 |  | 29.1(22.6-36.6) | 34.0(27.5-41.2) | 0.023 |  | <0.001 |  | <0.001 |
| *Southern Africa* | 45.2(43.6-46.9) | 50.1(48.8-51.4) | <0.001 |  | 66.2(64.6-67.8) | 65.8(64.6-67.0) | 0.345 |  | <0.001 |  | <0.001 |
| *Western Africa* | 54.4(53.3-55.5) | 48.6(47.5-49.6) | <0.001 |  | 68.4(67.3-69.4) | 61.9(60.9-62.9) | <0.001 |  | <0.001 |  | <0.001 |
| **BMI (kg/m^2^), mean±SD** | **25.8±13.6** | **30.3±15.6** | **<0.001** |  | **25.2±12.2** | **29.9±16.9** | **<0.001** |  |  |  |  |
| *Underweight (<18.5)* | 28.4(26.0-31.0) | 20.2(17.9-22.7) | <0.001 |  | 46.1(43.4-48.9) | 32.5(29.7-35.4) | <0.001 |  | <0.001 |  | <0.001 |
| *Normal weight (18.5-24.9)* | 39.9(38.7-41.1) | 33.4(32.2-34.6) | <0.001 |  | 58.5(57.3-59.7) | 49.3(48.0-50.6) | <0.001 |  | <0.001 |  | <0.001 |
| *Over weight (25.0-29.9)* | 60.9(59.2-62.6) | 51.0(49.5-52.5) | <0.001 |  | 77.4(75.9-78.9) | 67.3(65.9-68.7) | <0.001 |  | <0.001 |  | <0.001 |
| *Obese (≥30)* | 72.4(69.9-74.7) | 62.5(61.2-63.8) | <0.001 |  | 85.9(83.9-87.7) | 77.4(76.3-78.5) | <0.001 |  | <0.001 |  | <0.001 |
| Overall crude proportion | 48.7(47.9-49.6) | 47.3(46.6-48.1) | 0.02 |  | 65.7(64.9-66.5) | 62.5(61.7-63.2) | <0.001 |  | <0.001 |  | <0.001 |
| **Age-adjusted proportion of hypertension** | **37.7(36.2-39.2)** | **33.3(32.2-34.5)** | **-** |  | **57.4(55.5-59.3)** | **51.1(49.6-52.6)** | **-** |  | **-** |  | **-** |

*Note -* **Proportion** *was estimated as a function of the frequency of hypertension and the category total*

*BMI- Body mass index*

*P^1^ – Compares prevalence in men between hypertension definition 1 and 2*

*P^2^ – Compares prevalence in women between hypertension definition 1 and 2*

**Table S4. Association between obesity and hypertension adjusted for sex, age and country of residence in the Entire** Harmonized Dataset (EHD)

|  | **Hypertension defined as ≥140/90 mmHg** | | | | | | | |  | **Hypertension defined as ≥130/80 mmHg** | | | | | | | |
| --- | --- | --- | --- | --- | --- | --- | --- | --- | --- | --- | --- | --- | --- | --- | --- | --- | --- |
|  | **Combined sample** |  |  | **Male sample** |  |  | **Female sample** |  |  | **Combined sample** |  |  | **Male sample** |  |  | **Female sample** |  |
|  | **N=30044** |  |  | **n=12921** |  |  | **n=17123** |  |  | **N=30044** |  |  | **n=12921** |  |  | **n=17123** |  |
| **Factor** | **aOR(95%CI)** | **P** |  | **aOR(95%CI)** | **P** |  | **aOR(95%CI)** | **P** |  | **aOR(95%CI)** | **P** |  | **aOR(95%CI)** | **P** |  | **aOR(95%CI)** | **P** |
| **All age groups** | 2.4(2.2-2.5) | <0.001 |  | 2.8(2.5-3.2) | <0.001 |  | 2.2(2.0-2.4) | <0.001 |  | 2.5(2.3-2.6) | <0.001 |  | 3.0(2.6-3.6) | <0.001 |  | 2.3(2.1-2.5) | <0.001 |
| **Sex** *(male)* | 1.3(1.2-1.3) | <0.001 |  |  |  |  |  |  |  | 1.4(1.3-1.5) | <0.001 |  |  |  |  |  |  |
| **Age group** |  |  |  |  |  |  |  |  |  |  |  |  |  |  |  |  |  |
| *<40* | - | - |  | - | - |  | - | - |  | - | - |  | - | - |  | - | - |
| *40-44* | 2.0(1.8-2.2) | <0.001 |  | 2.0(1.7-2.3) | <0.001 |  | 2.1(1.8-2.4) | <0.001 |  | 1.7(1.6-1.9) | <0.001 |  | 1.7(1.5-2.0) | <0.001 |  | 1.7(1.5-1.9) | <0.001 |
| *45-49* | 2.7(2.5-3.0) | <0.001 |  | 2.2(1.9-2.6) | <0.001 |  | 3.2(2.8-3.6) | <0.001 |  | 2.1(2.0-2.3) | <0.001 |  | 1.8(1.6-2.1) | <0.001 |  | 2.4(2.1-2.7) | <0.001 |
| *50-54* | 3.8(3.5-4.2) | <0.001 |  | 3.1(2.7-3.6) | <0.001 |  | 4.5(4.0-5.1) | <0.001 |  | 3.0(2.7-3.2) | <0.001 |  | 2.4(2.1-2.7) | <0.001 |  | 3.5(3.1-3.9) | <0.001 |
| *55-59* | 5.0(4.6-5.5) | <0.001 |  | 4.0(3.5-4.6) | <0.001 |  | 5.9(5.3-6.7) | <0.001 |  | 3.6(3.3-4.0) | <0.001 |  | 3.0(2.6-3.5) | <0.001 |  | 4.2(3.7-4.7) | <0.001 |
| *60-64* | 7.3(6.5-8.1) | <0.001 |  | 6.5(5.4-7.6) | <0.001 |  | 8.1(6.9-9.4) | <0.001 |  | 4.9(4.3-5.5) | <0.001 |  | 4.2(3.5-5.0) | <0.001 |  | 5.5(4.7-6.4) | <0.001 |
| *≥65* | 11.4(10.2-12.8) | <0.001 |  | 8.8(7.4-10.3) | <0.001 |  | 14.1(12.1-16.5) | <0.001 |  | 8.4(7.4-9.6) | <0.001 |  | 6.3(5.2-7.6) | <0.001 |  | 10.7(9.0-12.7) | <0.001 |
| **Country** |  |  |  |  |  |  |  |  |  |  |  |  |  |  |  |  |  |
| *Burkina Faso* | 0.1(0.1-0.2) | <0.001 |  | 0.2(0.1-0.2) | <0.001 |  | 0.1(0.1-0.2) | <0.001 |  | 0.2(0.2-0.2) | <0.001 |  | 0.2(0.2-0.2) | <0.001 |  | 0.1(0.1-0.1) | <0.001 |
| *Cameroon* | 0.4(0.3-0.6) | <0.001 |  | 0.4(0.2-0.9) | 0.018 |  | 0.4(0.3-0.7) | <0.001 |  | 0.4(0.2-0.5) | <0.001 |  | 0.6(0.3-1.3) | 0.215 |  | 0.3(0.2-0.5) | <0.001 |
| *Ghana* | 0.7(0.6-0.8) | <0.001 |  | 0.6(0.5-0.7) | <0.001 |  | 0.8(0.7-0.8) | <0.001 |  | 0.6(0.6-0.7) | <0.001 |  | 0.6(0.5-0.6) | <0.001 |  | 0.7(0.6-0.8) | <0.001 |
| *Guinea* | 1.1(0.7-1.8) | 0.747 |  | 0.4(0.2-1.0) | 0.058 |  | 1.7(0.9-3.2) | 0.107 |  | 1.2(0.7-2.1) | 0.590 |  | 0.4(0.2-1.0) | 0.057 |  | 2.0(0.9-4.3) | 0.086 |
| *Kenya* | 0.2(0.2-0.3) | <0.001 |  | 0.2(0.2-0.2) | <0.001 |  | 0.3(0.2-0.3) | <0.001 |  | 0.4(0.3-0.4) | <0.001 |  | 0.3(0.2-0.3) | <0.001 |  | 0.4(0.4-0.5) | <0.001 |
| *Mozambique* | 0.4(0.3-0.5) | <0.001 |  | 0.4(0.2-0.6) | 0.000 |  | 0.4(0.3-0.6) | <0.001 |  | 0.5(0.4-0.6) | <0.001 |  | 0.5(0.3-0.7) | 0.001 |  | 0.5(0.4-0.7) | <0.001 |
| *Namibia* | 0.4(0.3-0.5) | <0.001 |  | 0.5(0.3-0.8) | 0.005 |  | 0.4(0.3-0.6) | <0.001 |  | 0.6(0.5-0.7) | <0.001 |  | 0.6(0.4-0.9) | 0.020 |  | 0.6(0.5-0.7) | <0.001 |
| *South Africa* | 0.6(0.6-0.6) | <0.001 |  | 0.5(0.4-0.5) | <0.001 |  | 0.7(0.6-0.8) | <0.001 |  | 0.7(0.6-0.7) | <0.001 |  | 0.6(0.5-0.7) | <0.001 |  | 0.7(0.6-0.8) | <0.001 |
| *Sudan* | 0.1(0.1-0.2) | <0.001 |  | 0.1(0.0-0.1) | 0.000 |  | 0.2(0.1-0.3) | <0.001 |  | 0.3(0.2-0.4) | <0.001 |  | 0.2(0.1-0.3) | <0.001 |  | 0.4(0.3-0.6) | <0.001 |
| *Tanzania* | 1.2(1.0-1.4) | 0.120 |  | 0.8(0.6-1.1) | 0.272 |  | 1.5(1.1-2.0) | 0.003 |  | 1.3(1.1-1.4) | 0.020 |  | 1.0(0.7-1.5) | 0.882 |  | 1.6(1.1-2.2) | 0.004 |
| *Uganda* | 0.8(0.7-1.0) | 0.031 |  | 0.8(0.6-1.0) | 0.050 |  | 0.9(0.7-1.1) | 0.244 |  | 1.1(0.9-1.3) | 0.289 |  | 1.0(0.8-1.3) | 0.905 |  | 1.1(0.9-1.4) | 0.252 |
| *Zambia* | 0.4(0.3-0.6) | <0.001 |  | 0.3(0.2-0.5) | 0.000 |  | 0.5(0.3-0.9) | 0.021 |  | 0.7(0.5-0.9) | 0.001 |  | 0.6(0.4-0.9) | 0.007 |  | 0.7(0.5-1.1) | 0.139 |
| *Nigeria* | - | - |  | - | - |  | - | - |  | - | - |  | - | - |  | - | - |

*aOR- Adjusted Odds Ratio; CI- Confidence interval*

**Table S5: Individual Project Participant Characteristics**

| **Project** | **Countries** | **Participants Inclusion Criteria** | |
| --- | --- | --- | --- |
| **AWI-Gen** | Burkina Faso Ghana  Kenya  South Africa | Male and Female ~ 50:50  40-60 years old  Population cross-section from communities  (Retrospective nested-case controls for cardiometabolic disease outcomes) | |
|  |  | **Cases (Inclusion criteria)** | **Controls (Inclusion criteria)** |
| **DM Group** | Cameroon  Guinea  Malawi  Nigeria  South Africa  Tanzania  Uganda | Male and Female  >25 years old  Diabetes: Fasting plasma glucose>7.0 mmol/l, or Random Plasma Glucose >11.1mmol or 2hr plasma glucose >11.1mmol  Oral/insulin treatment | Population based controls  >18 years  Not diabetic |
| **Kidney Group** | Ghana  Ethiopia Kenya  Nigeria | Male and Female  0-74 year  Diabetic CKD  Hypertensive CKD  Biopsy proven FSGS  Biopsy proven Minimal Change Disease  Biopsy proven Membranous Nephropathy  Childhood/Adolescent onset Steroid resistant Nephrotic Syndrome  HIV CKD  Sickle Cell Disease CKD  CKD of Unknown aetiology | Healthy individuals from communities and individuals attending ambulatory clinics or hospitalized patients with no evidence of kidney disease and no systemic condition known to be a common cause of kidney disease and eGFR>60ml/min/1.73m^2^ and random ACR mg/mmol of <3.5 mg/mmol in a female; <2.5 mg/mmol in a male)  Diabetes Controls  Hypertension Controls  HIV Controls  Sickle Cell Disease Controls  CKD of Unknown aetiology  General Population Controls |
| **RHDGen** | Kenya  Mozambique  Namibia  Nigeria  South Africa  Sudan  Uganda  Zambia | Male and Female  Echocardiographically confirmed cases of Rheumatic heart disease  Paediatric and adult cases (no age limit) | Male and Female  No valvular heart disease by echocardiography |
| **SIREN** | Ghana  Nigeria | Male and Female, 18- 100 years old  Stroke: Cranial CT/MRI confirmed first stroke episode | Unrelated subjects  No history of stroke, with or without CVD risk factors  Sex, age and ethnicity matched  >18 years  Male and Female recruited from communities during outreaches/community meetings |

*DM- Diabetes mellitus; CKD: Chronic Kidney Disease; FSGS- Focal segmental glomerulosclerosis; eGFR- estimated Glomerular filtration rate; CT- Computed Tomography; MRI- Magnetic Resonance imaging CVD- Cardiovascular Diseases; RHD- Rheumatic heart disease; SIREN- Stroke Investigative Research &Educational Network*

Table S6. Distribution of participants and crude prevalence of hypertension and obesity within each study according to country

|  |  |  |  | **Disease condition** | | |
| --- | --- | --- | --- | --- | --- | --- |
| **Country** | **Study** | **Number of**  **participants** | **Percentage §** | **Hypertension defined as ≥140/90 mmHg ƺ** | **Hypertension defined as ≥130/80 mmHg ƺ** | **Obese ƺ** |
| Burkina Faso | AWIGEN | 2082 | 100.0 | 16.4 | 39.7 | 2.0 |
|  | **Sub Total** | **2082** |  |  |  |  |
| Cameroon | DM GROUP | 117 | 100.0 | 55.6 | 75.2 | 45.3 |
|  | **Sub Total** | **117** |  |  |  |  |
| Ghana | AWIGEN | 1834 | 27.5 | 25.2 | 55.0 | 3.1 |
|  | KIDNEY GROUP | 3363 | 50.5 | 58.3 | 78.4 | 21.2 |
|  | SIREN | 1469 | 22.0 | 75.8 | 89.7 | 22.2 |
|  | **Sub Total** | **6666** |  |  |  |  |
| Guinea | DM GROUP | 74 | 100.0 | 64.9 | 85.1 | 14.9 |
|  | **Sub Total** | **74** |  |  |  |  |
| Kenya | AWIGEN | 2001 | 78.3 | 27.0 | 53.3 | 19.8 |
|  | KIDNEY GROUP | 20 | 0.8 | 50.0 | 50.0 | 10.0 |
|  | RHDGEN | 534 | 20.9 | 8.4 | 59.9 | 11.6 |
|  | **Sub Total** | **2555** |  |  |  |  |
| Mozambique | RHDGEN | 358 | 100.0 | 24.9 | 57.0 | 22.6 |
|  | **Sub Total** | **358** |  |  |  |  |
| Namibia | RHDGEN | 481 | 100.0 | 23.5 | 58.4 | 22.0 |
|  | **Sub Total** | **481** |  |  |  |  |
| Nigeria | DM GROUP | 774 | 9.9 | 6.7 | 12.1 | 3.6 |
|  | KIDNEY GROUP | 4581 | 58.5 | 54.0 | 76.7 | 18.4 |
|  | RHDGEN | 129 | 1.6 | 11.6 | 50.4 | 13.2 |
|  | SIREN | 2342 | 29.9 | 77.5 | 90.5 | 15.9 |
|  | **Sub Total** | **7826** |  |  |  |  |
| South Africa | AWIGEN | 5140 | 65.2 | 51.9 | 77.6 | 34.6 |
|  | DM GROUP | 1622 | 20.6 | 65.6 | 76.1 | 56.9 |
|  | RHDGEN | 1118 | 14.2 | 33.9 | 70.5 | 31.1 |
|  | **Sub Total** | **7880** |  |  |  |  |
| Tanzania | DM GROUP | 572 | 100.0 | 70.5 | 88.5 | 28.8 |
|  | **Sub Total** | **572** |  |  |  |  |
| Uganda | DM GROUP | 498 | 63.8 | 74.1 | 91.6 | 34.9 |
|  | RHDGEN | 282 | 36.2 | 14.9 | 57.8 | 13.1 |
|  | **Sub Total** | **780** |  |  |  |  |
| Zambia | RHDGEN | 313 | 100.0 | 16.0 | 52.7 | 5.8 |
|  | **Sub Total** | **313** |  |  |  |  |
| Sudan | RHDGEN | 340 | 100.0 | 8.2 | 53.5 | 11.5 |
|  | **Sub Total** | **340** |  |  |  |  |
| **Overall for Studies** | **AWIGEN** | **11057** | 36.8 | **36.3** | **62.3** | **20.6** |
|  | **DM GROUP** | **3657** | 12.2 | **61.7** | **79.4** | **40.8** |
|  | **KIDNEY GROUP** | **7964** | 26.5 | **55.8** | **77.3** | **19.6** |
|  | **RHDGEN** | **3555** | 11.8 | **21.4** | **61.0** | **19.9** |
|  | **SIREN** | **3811** | 12.7 | **76.9** | **90.2** | **18.3** |
|  | **Grand Total** | **30044** |  |  |  |  |

*§ - Percentages were based on the country sub-total while study overall percentages were based on the Grand totals*

**ƺ -** *Percentages were based on the number of participants within study per country while study overall percentages were based on the number of participants per study*

*DM- Diabetes mellitus; RHD- Rheumatic heart disease; SIREN- Stroke Investigative Research &Educational Network*

**Table S7. Phenotype Measurement across studies and harmonization strategies**

| **Phenotype** | **Data capturing and Measurement procedures across study** | **Summary of Harmonization procedures and final variable definition** |
| --- | --- | --- |
| Age | Age in years was measured across studies. | Age was harmonized across studies as number of years lived as at the time of data collection. This was recoded in ten years interval at the point of analysis. |
| Sex | Biological sex (Male and Female) was reported for every participants across studies | Sex was harmonized across studies as the biological sex (Male or Female). This was coded 1 for male and 2 for female at the point of data analysis. |
| Country of residence | Participants’ country of residence was reported for participants across studies. | Country of residence was harmonized as the country in which the participant was recruited into the study. |
| Height | Height was measured in meters across studies except one (the Kidney group) where height was measured in centimeters. | Height was harmonized across studies by converting measurements in centimeters to meters (i.e 100cm equals to 1m). |
| Weight | Weight was measured in kilograms for participants (wearing light dresses and without shoes on) across studies |  |
| Body mass index (BMI) | Body Mass Index (BMI) was estimated as weight in kilograms per height in meters squared across studies. | BMI was harmonized across studies as weight in kilograms per height in meters squared.  BMI (kg/m^2^), was categorized as  Underweight (<18.5), Normal weight (18.5-24.9), Over weight (25.0-29.9) and Obese (≥30). |
| Blood pressure (Systolic and Diastolic) | Blood pressure was measured three times for each participant across all studies using appropriate tools and sphygmomanometer cuff sizes according to standard procedures.^16^ | Baseline blood pressure was harmonized across studies as an average of at least two (preferably the last two) of the baseline readings per participant.^16^ |
| Hypertension | The following details were captured for participants across studies:   1. Previous diagnosis of hypertension by a health professional, 2. Current systolic BP≥140mmHg or diastolic BP≥90 3. Use of antyhypertensive medications | Hypertension  data were harmonized and defined as sustained systolic  BP≥140 mmHg or diastolic BP≥90 mmHg, history  of hypertension, or taking antihypertensive medications. |


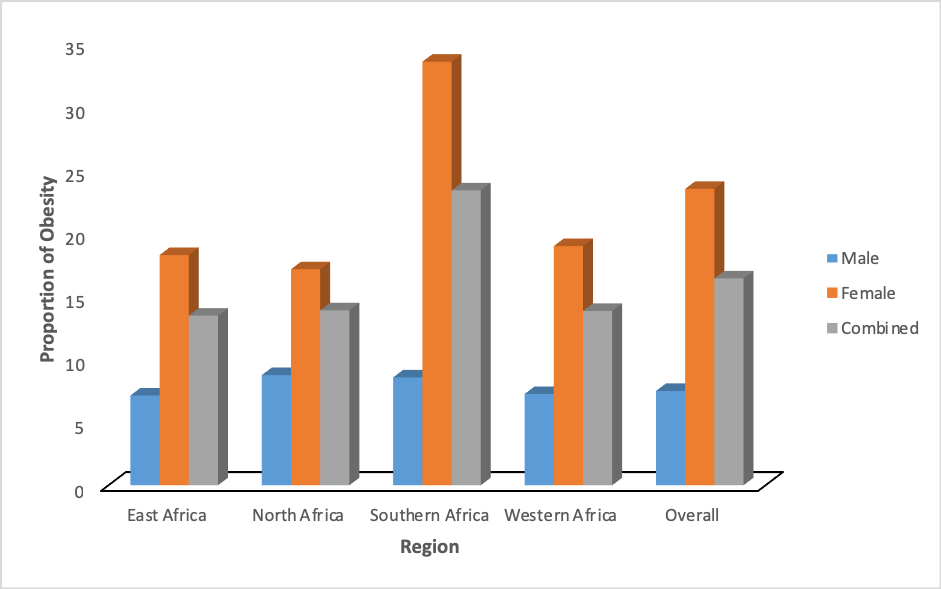


**Figure S1.** Regional differences in age-adjusted proportion of obesity using only the disease-free population control (PC) dataset (for male, female and the combined samples)


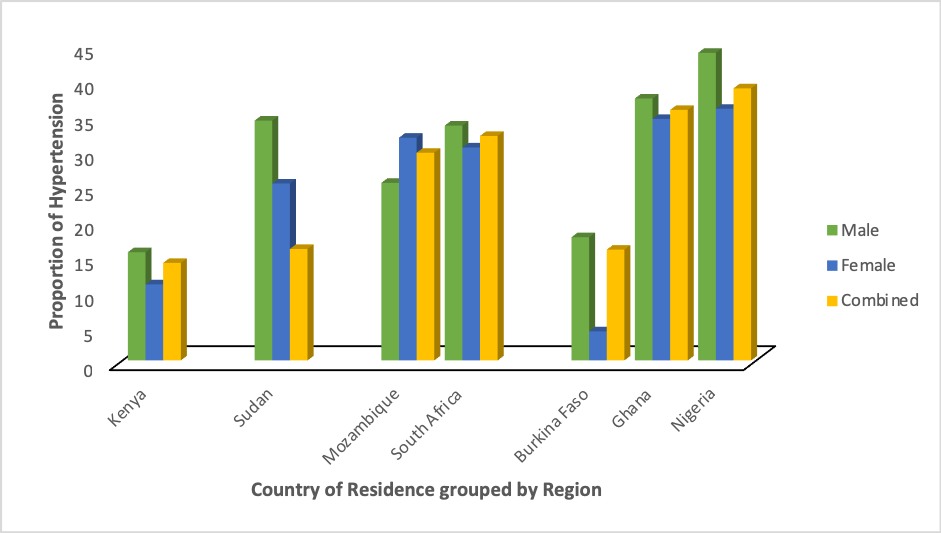


**Figure S2.** Age-adjusted proportion of hypertension using only the population control dataset (for male, female and the combined samples) across countries grouped by Region

**
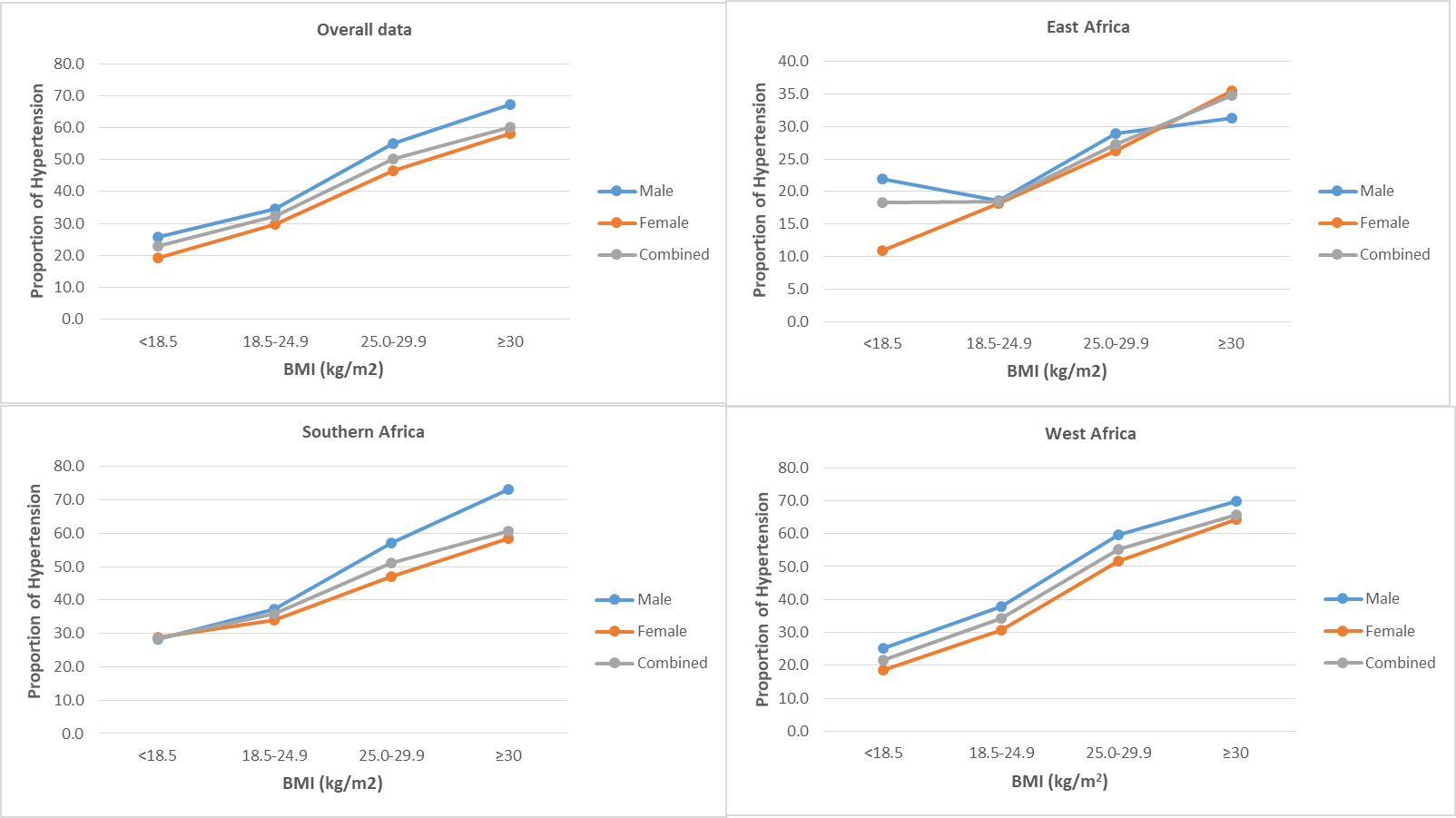
**

**Figure S3.** Proportion of hypertension (≥140/90 mm Hg) across BMI categories groups (for male, females and the combined samples) in the Population Control (PC) dataset


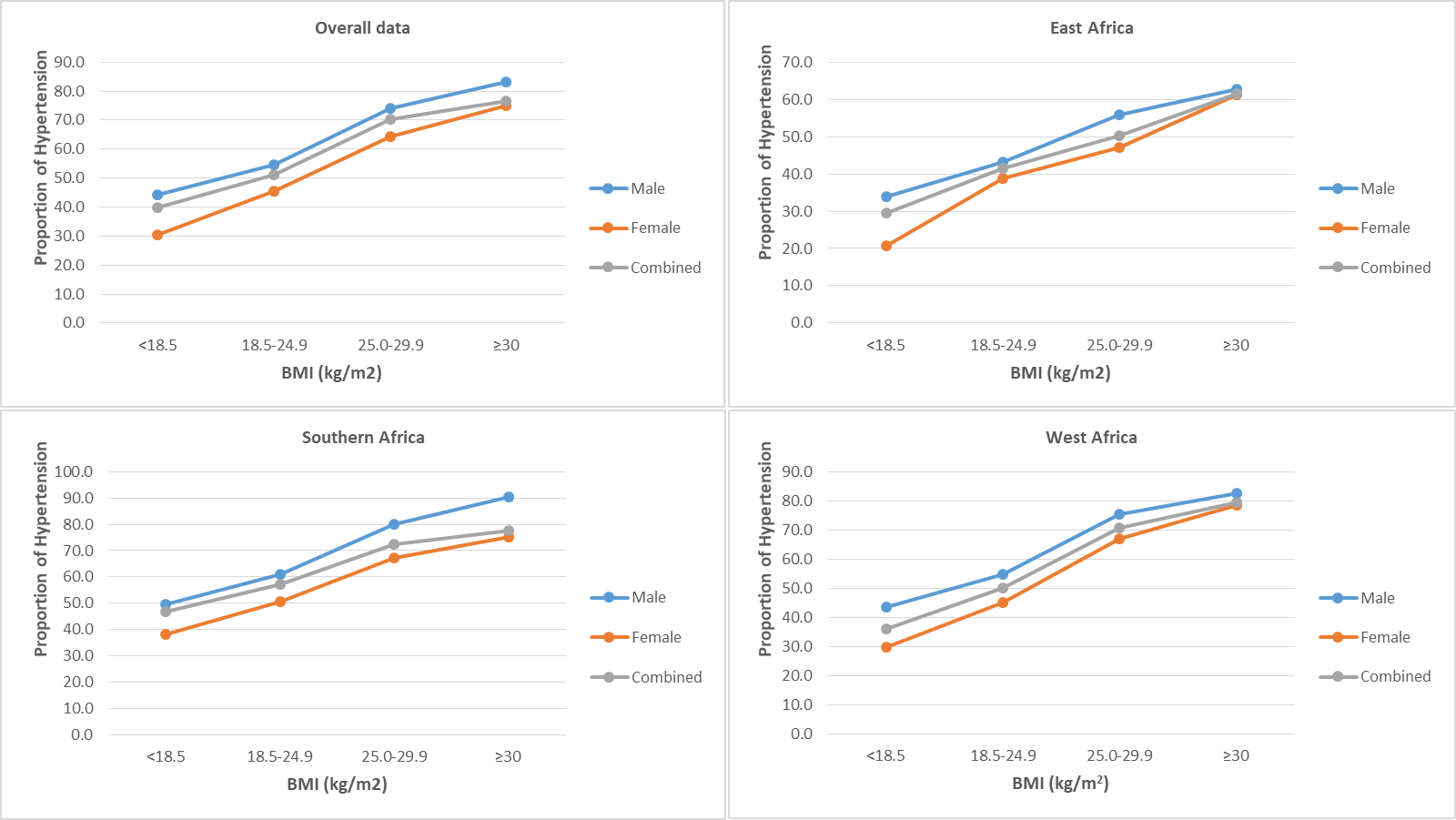


**Figure S4.** Proportion of hypertension (≥130/80 mm Hg) across BMI categories groups (for male, females and the combined samples) in the Population Control (PC) dataset

**
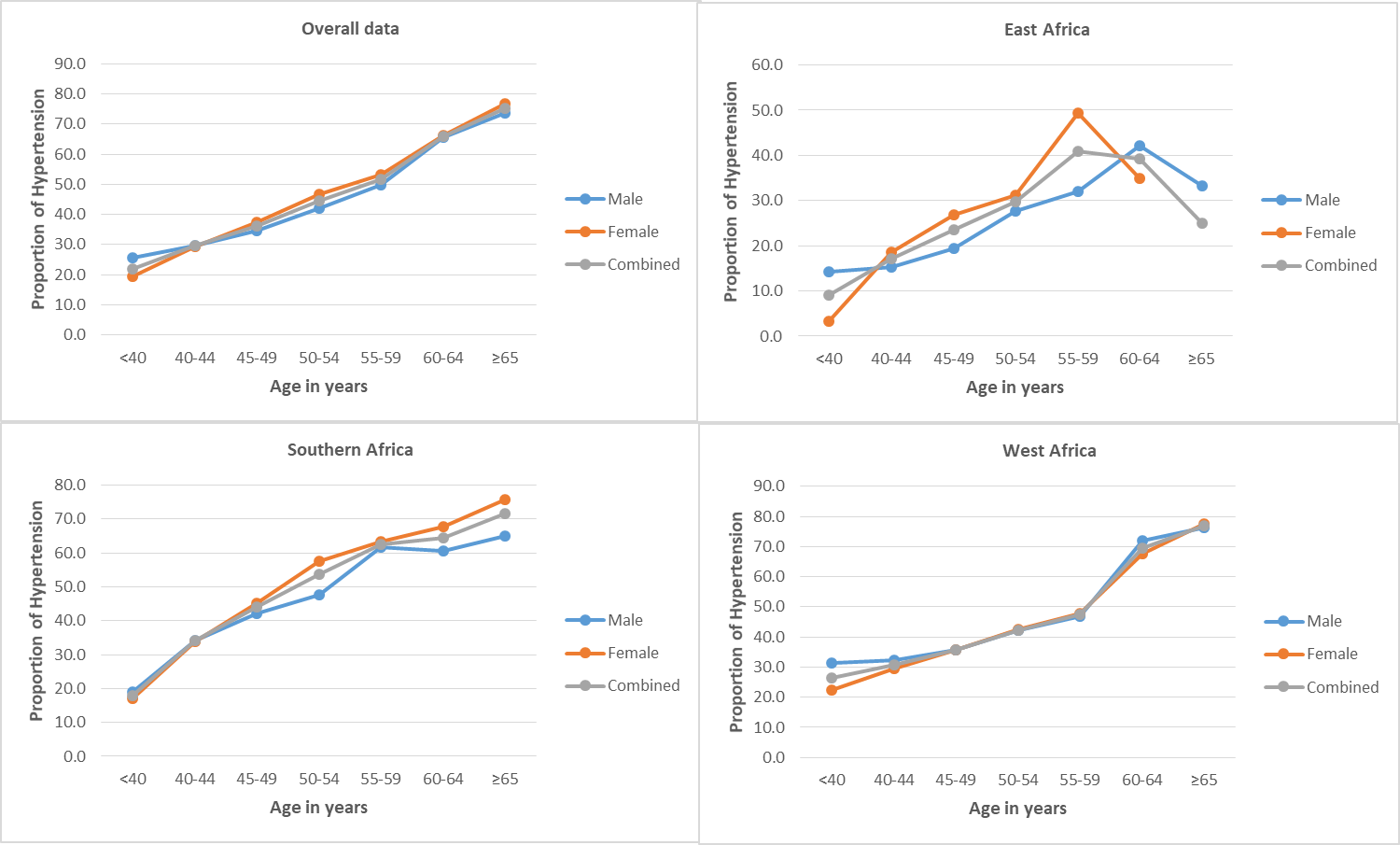
**

**Figure S5. Proportion of hypertension defined as ≥140/90 mm Hg across age groups (for male, female and the combined samples) in the Population Control (PC) dataset**

**
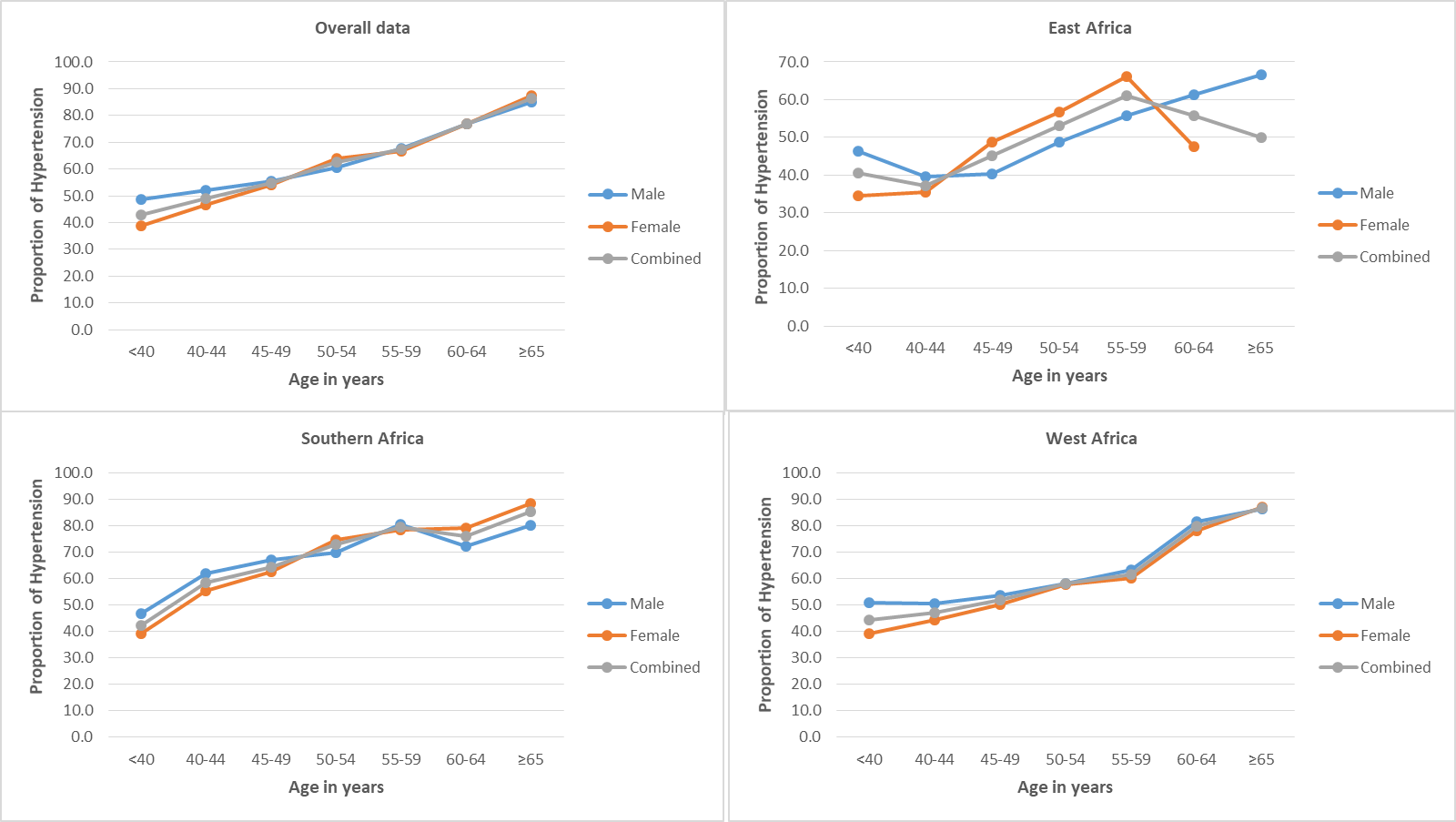
**

**Figure S6. Proportion of hypertension (≥130/80 mm Hg) across age groups (for male, female and the combined samples) in the Population Control (PC) dataset**

*
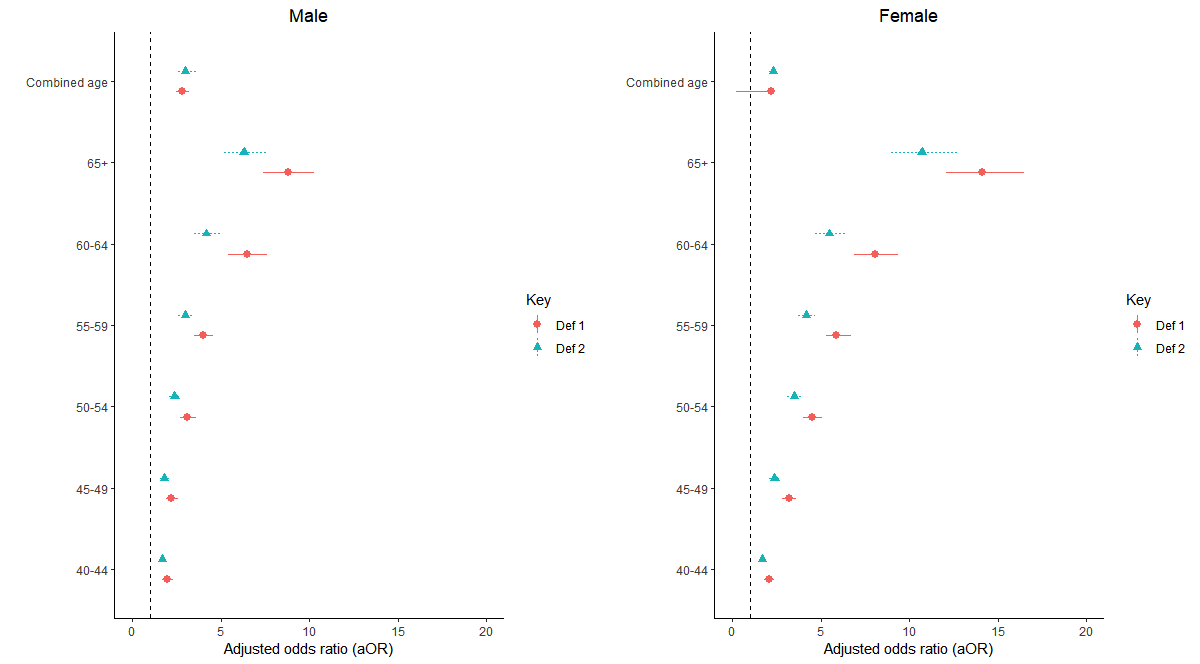
*

*def1- Hypertension defined as ≥140/90 mmHg; def2- Hypertension defined as ≥130/80 mmHg; Age group <40years was the reference age group in the analysis*

**Figure S7. Adjusted Odds of Hypertension in obesity (defined by ≥140/90 mmHg and ≥130/80 mmHg) across age strata, stratified by sex of participants in CHAIR Entire Harmonized Data (EHD)**
