# Supplementary figures and images for: Regional Patterns and Association Between Obesity and Hypertension in Africa: Evidence From the H3Africa CHAIR Study
Source: Hypertension. 2020 Mar 16;75(5):1167–78. doi: 10.1161/HYPERTENSIONAHA.119.14147 (PMC7176339; doi:10.1161/HYPERTENSIONAHA.119.14147)

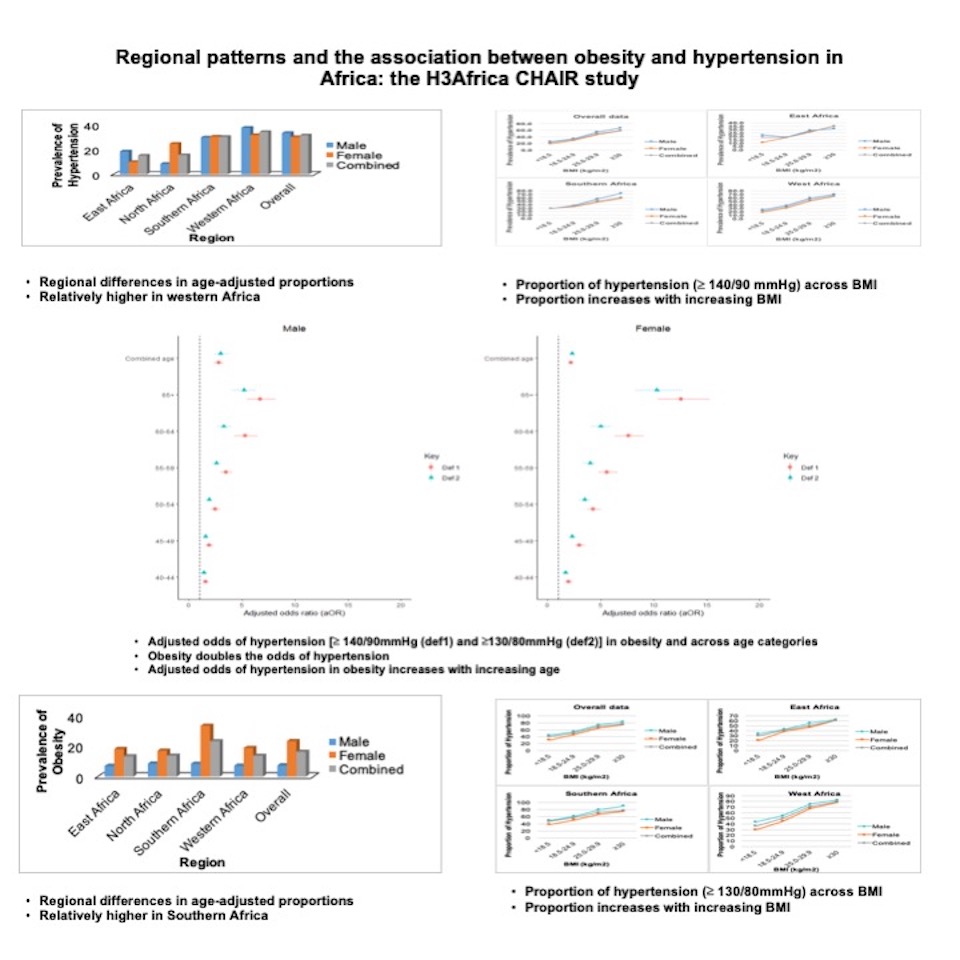

Supplement: Supplementary file 2 [file hyp-75-1167-s002.jpg]
