# Supplementary material for: Regional Patterns and Association Between Obesity and Hypertension in Africa: Evidence From the H3Africa CHAIR Study
Source: Hypertension. 2020 Mar 16;75(5):1167–78. doi: 10.1161/HYPERTENSIONAHA.119.14147 (PMC7176339; doi:10.1161/HYPERTENSIONAHA.119.14147)
Supplement: Supplementary file 3 [file hyp-75-1167-s003.doc]

**Full list of Authors**

**Authors:**

Onoja M. Akpa, Ph.D.^1-3,5^, Felix Made, M.Sc.^4,5^, Akinlolu Ojo, MD, Ph.D.^6^, Bruce Ovbiagele MD, FAAN^7^, Dwomoa Adu MD^8^, Ayesha A. Motala, MD^9^, Bongani M. Mayosi, MBChB, DPhil^10&^, Sally N. Adebamowo, MD, ScD^11^, Mark E. Engel, MPH, Ph.D.^12^, Bamidele Tayo, PhD^13^, Charles Rotimi, Ph.D.^14^, Babatunde Salako, MD^1^ Rufus Akinyemi MBBS, Ph.D.^1^, Mulugeta Gebregziabher, Ph.D.^15^, Fred Sarfo MBBS, Ph.D.^16^, Kolawole Wahab MBBS, MPH^17^, Godfred Agongo, MPhil^18^, Marianne Alberts, Ph.D.^19^, Stuart A Ali, Ph.D.^5^, Gershim Asiki, Ph.D.^20^, Romuald P Boua, M.Sc.^21^, F Xavier Gómez-Olivé, Ph.D.^22^, Felistas Mashinya, Ph.D.^19^, Lisa Micklesfield, Ph.D.^23^, Shukri F Mohamed, PharmD, MPH^20^, Engelbert A Nonterah, MBchB,M.Sc.^18^, Shane A Norris, Ph.D.^23^, Hermann Sorgho, Ph.D.^21^, Stephen Tollman, MPH, Ph.D.^22^, Rulan S. Parekh, MD,MS^24^, Chisala Chisala^12^, Kenneth Ekuro, Ph.D.^25^, Salina P. Waddy, MD^26^, Emmanuel Peprah Ph.D.^27^, George A. Mensah MD^28^, Ken Wiley Ph.D.^29^, Jennifer Troyer Ph.D^30^, Michèle Ramsay, Ph.D.^5#^ Mayowa O. Owolabi MBBS, MSc, DrM, FAAN, FAS^1*#^,as members of the CVD Working Group of the H3Africa Consortium

^&^ Deceased

*^#^ Joint senior authors*

**Funding and Acknowledgement**

The harmonization process for the CHAIR consortium is supported by the administrative National Institutes of Health (NIH) supplement U54HG007479-03S1 to the SIREN study for the benefit of the H3Africa CVD Working Group. SIREN is funded by the NIH (Grant U54HG007479; NINDS/NHGRI/NIEHS); Systematic Investigation of Blacks with Stroke using Genomics (SIBS Genomics) NIH (NINDS/NHGRI), R01NS107900; African Neurobiobank for Precision Stroke Medicine - (ELSI) Project NIH 1U01HG010273; Africa-UK Collaboration for the genetic Epidemiology of Stroke (ACES) is funded by the Academy of Medical Sciences Global Challenges Research Fund Networking Grant Scheme (GCRFNGR2\10190). The AWI-Gen Collaborative Centre is funded by the NIH (NHGRI; NICHD; OD) (Grant U54HG006938). The ACCME cohort is funded by the NIH (NHGRI grant U54HG006947). The H3Africa Kidney Disease Research Consortium if funded by the NIH (Grant U54HG006939). The DM Study (Burden, spectrum and aetiology of type 2 diabetes in sub-Saharan Africa) is funded by the Wellcome Trust (Grant No. WT 099316AIA). The RHDGen Network is funded by the Wellcome Trust (099313/Z/12/Z and 099313/B/12/Z). Charles Rotimi is supported by the intramural program of the NHGRI/NIH at the Center for Research on Genomics and Global Health (CRGGH); The CRGGH is also supported by NIDDK, CIT and the NIH Office of the Director. Rulan S. Parekh is funded by the Canada Research Chair in Chronic Kidney Disease Epidemiology. The authors acknowledge Jean-Tristan Brandenburg for assisting with the forest plots. The content is solely the responsibility of the authors and does not necessarily represent the official views of the National Institutes of Health and the Wellcome Trust.

**Role of the Funding Source:** The funders played no role in the data collection or interpretation of this study.

**Authors’ contributions**

Co-authors contributed to one or more of the following: study concept, design and/or data acquisition. OMA and FM are CHAIR statisticians who contributed to data harmonization and analysis. MOO, MR, OMA and FM contributed to interpretation of results and drafting of the manuscript. All authors contributed to critical revision of the manuscript and approval of the final draft.

**Disclosures**

None

**Affiliations:**

1. Center for Genomic and Precision Medicine, University of Ibadan (O.M.A., R.A., B.S., M.O.O.)
2. Department of Epidemiology and Medical Statistics, College of Medicine, University of Ibadan (O.M.A.)
3. Institute of Cardiovascular Diseases, College of Medicine, University of Ibadan (O.M.A.)
4. The Epidemiology and Surveillance Section, National Institute for Occupational Health, National Health Laboratory Services, Gauteng Region, South Africa (F.M.)
5. Sydney Brenner Institute for Molecular Bioscience and Division of Human Genetics, Faculty of Health Sciences, University of the Witwatersrand, Johannesburg, South Africa (O.M.A. F.M., S.A.A., M.R.)
6. Clinical research and global health initiatives, University of Arizona Health Sciences (A.O.)
7. Department of Neurology, University of California, San Francisco (B.O.)
8. School of Medicine and Dentistry, University of Ghana. P.O. Box 4236, Accra, Ghana (D.A.)
9. Department of Diabetes and Endocrinology, Nelson R. Mandela School of Medicine, University of KwaZulu-Natal, Durban, South Africa (A.A.M.)
10. Department of Medicine, Groote Schuur Hospital and University of Cape Town, Cape Town, South Africa (B.M.M.)
11. Department of Epidemiology and Public Health; and Greenebaum Comprehensive Cancer Center, University of Maryland School of Medicine, Baltimore, MD 21201 (S.N.A)
12. Division of Cardiology, Department of Medicine, University of Cape Town, Cape Town, South Africa (M.E.E., C.C.)
13. Department of Preventive Medicine and Epidemiology, Loyola University Chicago Stritch School of Medicine, Maywood, Illinois, United States of America (B.T.)

14. Center for Research on Genomics and Global Health, NHGRI, NIH, Bethesda, Maryland, USA (C.R.)

15. Department of Public Health Sciences, Medical University of South Carolina, Charleston, USA (M.G.)

16. Kwame Nkrumah University of Science and Technology, Kumasi, Ghana (F.S.)

17. Department of Medicine, University of Ilorin, Ilorin, Nigeria (K.W.)

18. Navrongo Health Research Centre, Navrongo, Ghana (G.A., E.A.N.)

19. Department of Pathology and Medical Science, School of Health Care Sciences, Faculty of Health Sciences, University of Limpopo, Polokwane, South Africa (M.A., F.M.)

20. African Population and Health Research Center, Nairobi, Kenya (G.A. S.F.M.)

21. Institut de Recherche en Sciences de la Sante, Clinical Research Unit of Nanoro, Burkina Faso (R.P.B., H.S.)

22. MRC/Wits Rural Public Health and Health Transitions Research Unit (Agincourt), School of Public Health, Faculty of Health Sciences, University of the Witwatersrand, Johannesburg 2193, South Africa (F.G., S.T.)

23. MRC/Wits Developmental Pathways for Health Research Unit, Faculty of Health Sciences, University of the Witwatersrand, Johannesburg, South Africa (L.M., S.A.N.)

24. Departments of Pediatrics, Medicine and Epidemiology, Hospital for Sick Children, University Health Network and University of Toronto (R.S.P.)

25. Center for Research on Genomics and Global Health, National Human Genome Research Institute, National Institutes of Health (K.E.)

26. Department of Neurology, Atlanta Veterans Affairs Medical Center, Decatur, Georgia, USA

27. New York University, College of Global Public Health, U.S.A. (E.P.)

28. Center for Translation Research and Implementation Science, National Heart, Lung, and Blood Institute, NIH, Bethesda, USA (G.A.M.)

29. Division of Genomic Medicine, National Human Genome Research Institute, National Institutes of Health, USA (K.W.)

30. Human Heredity and Health in Africa, Division of Genome Sciences, National Institutes of Health, USA (J.T.)
