# Supplementary material for: Regional Patterns and Association Between Obesity and Hypertension in Africa: Evidence From the H3Africa CHAIR Study
Source: Hypertension. 2020 Mar 16;75(5):1167–78. doi: 10.1161/HYPERTENSIONAHA.119.14147 (PMC7176339; doi:10.1161/HYPERTENSIONAHA.119.14147)
Supplement: Supplementary file 4 [file hyp-75-1167-s004.pdf]

Change of Authorship Form

(Must be completed and signed by ALL authors)

Please check all that apply

_____New author(s) have been added (in addition to this form, all new authors must complete the copyright transfer agreement and conflict of interest disclosure.

_____Change in order of authorship.

_____An author wishes to remove his/her name. An author's name may only be removed his/her own request and a letter signed by the author should accompany this form

Manuscript Number HYPE2019141

Manuscript Title Regional patterns and association between obesity and hypertension in Africa: E

Former Authorship

Please list ALL AUTHORS in the same order as the original submission. For more than 12, use an extra sheet.

Print Name	Print Name
Name (1) Onoja M. Akpa	Name (7) Bongani M. Mayosi
Name (2) Felix Made	Name (8) Sally N. Adebamowo
Name (3) Akinlolu Ojo	Name (9) Mark E. Engel
Name (4) Bruce Ovbiagele	Name (10) Charles Rotimi
Name (5) Dwomoa Adu	Name (11) Ken Wiley
Name (6) Ayesha A. Motala	Name (12) Jennifer Troyer

New Authorship

All authors must sign below agreeing to the changes in authorship. The authorship order must reflect the authorship order of the manuscript.

Name	Signature	Date
Name (1) Onoja M. Akpa	[signature]	22/01/2020
Name (2) Felix Made	F Made	23/01/20
Name (3) Akinlolu Ojo		
Name (4) Bruce Ovbiagele		
Name (5) Dwomoa Adu		
Name (6) Ayesha A. Motala		
Name (7) Bongani M. Mayosi	Deceased	
Name (8) Sally N. Adebamowo		
Name (9) Mark E. Engel		
Name (10) Bamidele Tayo	Bamidele Tayo Digitally signed by Bamidele Tayo DN: cn=Bamidele Tayo, o=Loyola University Chicago, ou, email=btayo@luc.edu, c=US Date: 2020.01.23 16:36:28 -06'00'	23-Jan-20
Name (11) Charles Rotimi		
Name (12) Babatunde Salako		

Please print form and fax to 214-706-1565.

Change of Authorship Form

(Must be completed and signed by ALL authors)

Please check all that apply

_____New author(s) have been added (in addition to this form, all new authors must complete the copyright transfer agreement and conflict of interest disclosure.

_____Change in order of authorship.

_____An author wishes to remove his/her name. An author's name may only be removed his/her own request and a letter signed by the author should accompany this form

Manuscript Number HYPE2019141

Manuscript Title Regional patterns and association between obesity and hypertension in Africa: E

Former Authorship

Please list ALL AUTHORS in the same order as the original submission. For more than 12, use an extra sheet.

**Print Name**		**Print Name**	
Name (1)	Onoja M. Akpa	Name (7)	Bongani M. Mayosi
Name (2)	Felix Made	Name (8)	Sally N. Adebamowo
Name (3)	Akinlolu Ojo	Name (9)	Mark E. Engel
Name (4)	Bruce Ovbiagele	Name (10)	Charles Rotimi
Name (5)	Dwomoa Adu	Name (11)	Ken Wiley
Name (6)	Ayesha A. Motala	Name (12)	Jennifer Troyer

New Authorship

All authors must sign below agreeing to the changes in authorship. The authorship order must reflect the authorship order of the manuscript.

	Name	Signature	Date
Name (1)	Onoja M. Akpa		22/01/2020
Name (2)	Felix Made		23/01/20
Name (3)	Akinlolu Ojo	Akinlolu Ojo	28/01/2020
Name (4)	Bruce Ovbiagele		
Name (5)	Dwomoa Adu	dwomoaadu	27/01/2020
Name (6)	Ayesha A. Motala		
Name (7)	Bongani M. Mayosi		
Name (8)	Sally N. Adebamowo		
Name (9)	Mark E. Engel		
Name (10)	Bamidele Tayo		
Name (11)	Charles Rotimi	Charleskotimi	
Name (12)	Babatunde Salako		

Please print form and fax to 214-706-1565.

Change of Authorship Form

(Must be completed and signed by ALL authors)

Please check all that apply

_____New author(s) have been added (in addition to this form, all new authors must complete the copyright transfer agreement and conflict of interest disclosure.

_____Change in order of authorship.

_____An author wishes to remove his/her name. An author's name may only be removed his/her own request and a letter signed by the author should accompany this form

Manuscript Number HYPE2019141

Manuscript Title Regional patterns and association between obesity and hypertension in Africa: E

Former Authorship

Please list ALL AUTHORS in the same order as the original submission. For more than 12, use an extra sheet.

**Print Name**		**Print Name**	
Name (1)	Onoja M. Akpa	Name (7)	Bongani M. Mayosi
Name (2)	Felix Made	Name (8)	Sally N. Adebamowo
Name (3)	Akinlolu Ojo	Name (9)	Mark E. Engel
Name (4)	Bruce Ovbiagele	Name (10)	Charles Rotimi
Name (5)	Dwomoa Adu	Name (11)	Ken Wiley
Name (6)	Ayesha A. Motala	Name (12)	Jennifer Troyer

New Authorship

All authors must sign below agreeing to the changes in authorship. The authorship order must reflect the authorship order of the manuscript.

Name (1)	Onoja M. Akpa	Signature		Date	22/01/2020
Name (2)	Felix Made	Signature	F. Made	Date	23/01/20
Name (3)	Akinlolu Ojo	Signature		Date	
Name (4)	Bruce Ovbiagele	Signature	B.Ovbiagele	Date	24/01/20
Name (5)	Dwomoa Adu	Signature		Date	
Name (6)	Ayesha A. Motala	Signature	[illegible]	Date	24/01/20
Name (7)	Bongani M. Mayosi	Signature	Deceased	Date	
Name (8)	Sally N. Adebamowo	Signature		Date	
Name (9)	Mark E. Engel	Signature		Date	
Name (10)	Bamidele Tayo	Signature		Date	
Name (11)	Charles Rotimi	Signature		Date	
Name (12)	Babatunde Salako	Signature		Date	

Please print form and fax to 214-706-1565.

Change of Authorship Form

(Must be completed and signed by ALL authors)

Please check all that apply

_____New author(s) have been added (in addition to this form, all new authors must complete the copyright transfer agreement and conflict of interest disclosure.

_____Change in order of authorship.

_____An author wishes to remove his/her name. An author's name may only be removed his/her own request and a letter signed by the author should accompany this form

Manuscript Number HYPE2019141

Manuscript Title Regional patterns and association between obesity and hypertension in Africa: E

Former Authorship

Please list ALL AUTHORS in the same order as the original submission. For more than 12, use an extra sheet.

**Print Name**		**Print Name**	
Name (1)	Onoja M. Akpa	Name (7)	Bongani M. Mayosi
Name (2)	Felix Made	Name (8)	Sally N. Adebamowo
Name (3)	Akinlolu Ojo	Name (9)	Mark E. Engel
Name (4)	Bruce Ovbiagele	Name (10)	Charles Rotimi
Name (5)	Dwomoa Adu	Name (11)	Ken Wiley
Name (6)	Ayesha A. Motala	Name (12)	Jennifer Troyer

New Authorship

All authors must sign below agreeing to the changes in authorship. The authorship order must reflect the authorship order of the manuscript.

	Name		Signature		Date
Name (1)	Onoja M. Akpa	Signature		Date	22/01/2020
Name (2)	Felix Made	Signature	F. Made	Date	23/01/20
Name (3)	Akinlolu Ojo	Signature		Date	
Name (4)	Bruce Ovbiagele	Signature	B.Ovbiagele	Date	24/01/20
Name (5)	Dwomoa Adu	Signature		Date	
Name (6)	Ayesha A. Motala	Signature	[illegible]	Date	24/01/20
Name (7)	Bongani M. Mayosi	Signature	Deceased	Date	
Name (8)	Sally N. Adebamowo	Signature		Date	
Name (9)	Mark E. Engel	Signature	M Engel	Date	28/01/2020
Name (10)	Bamidele Tayo	Signature		Date	
Name (11)	Charles Rotimi	Signature		Date	
Name (12)	Babatunde Salako	Signature	B. Salako	Date	28/01/2020

Please print form and fax to 214-706-1565.

Change of Authorship Form

(Must be completed and signed by ALL authors)

Please check all that apply

__✔__ New author(s) have been added (in addition to this form, all new authors must complete the copyright transfer agreement and conflict of interest disclosure.

_____ Change in order of authorship.

_____ An author wishes to remove his/her name. An author's name may only be removed his/her own request and a letter signed by the author should accompany this form

Manuscript Number HYPE201914147-T1

Manuscript Title Regional patterns and association between obesity and hypertension in Africa: Evidence from the H3Africa CHAIR study

Former Authorship

Please list ALL AUTHORS in the same order as the original submission. For more than 12, use an extra sheet.

Print Name		Print Name	
Name (1)	Onoja M. Akpa	Name (7)	Bongani M. Mayosi
Name (2)	Felix Made	Name (8)	Sally N. Adebamowo
Name (3)	Akinlolu Ojo	Name (9)	Mark E. Engel
Name (4)	Bruce Ovbiagele	Name (10)	Charles Rotimi
Name (5)	Dwomoa Adu	Name (11)	Ken Wiley
Name (6)	Ayesha A. Motala	Name (12)	Jennifer Troyer

New Authorship

All authors must sign below agreeing to the changes in authorship. The authorship order must reflect the authorship order of the manuscript.

	Name	Signature	Date
Name (1)	Onoja M. Akpa	O.M. Akpa	22/01/20
Name (2)	Felix Made	F. Made	23/01/20
Name (3)	Akinlolu Ojo		
Name (4)	Bruce Ovbiagele		
Name (5)	Dwomoa Adu		
Name (6)	Ayesha A. Motala		
Name (7)	Bongani M. Mayosi	Deceased	
Name (8)	Sally N. Adebamowo	SallyA	01/24/2020
Name (9)	Mark E. Engel		
Name (10)	Bamidele Tayo		
Name (11)	Charles Rotimi		
Name (12)	Babatunde Salako		

Please print form and fax to 214-706-1565.

Change of Authorship Form

(Must be completed and signed by ALL authors)

Please check all that apply

__✔__ New author(s) have been added (in addition to this form, all new authors must complete the copyright transfer agreement and conflict of interest disclosure.

_____ Change in order of authorship.

_____ An author wishes to remove his/her name. An author's name may only be removed his/her own request and a letter signed by the author should accompany this form

Manuscript Number HYPE2019141

Manuscript Title Regional patterns and association between obesity and hypertension in Africa: E

Former Authorship

Please list ALL AUTHORS in the same order as the original submission. For more than 12, use an extra sheet.

**Print Name**		**Print Name**	
Name (1)	Michèle Ramsay	Name (7)	
Name (2)	Mayowa O. Owolabi	Name (8)	
Name (3)		Name (9)	
Name (4)		Name (10)	
Name (5)		Name (11)	
Name (6)		Name (12)	

New Authorship

All authors must sign below agreeing to the changes in authorship. The authorship order must reflect the authorship order of the manuscript.

Name		Signature	Date
Name (1)	Rufus Akinyemi		
Name (2)	Mulugeta Gebregziabher	M.Gebregziabher	24/01/20
Name (3)	Fred Sarfo	FSSarfo	25/01/2020
Name (4)	Kolawole Wahab		
Name (5)	Godfred Agongo	Godfred Aagongo	24/01/20
Name (6)	Marianne Alberts		
Name (7)	Stuart Ali		
Name (8)	Gershim Asiki	[illegible]	28/01/2020
Name (9)	Romuald Boua		
Name (10)	F Xavier Gómez-Olivé,		
Name (11)	Felistas Mashinya		
Name (12)	Lisa Micklesfield	Micklesfield	24-01-20

Please print form and fax to 214-706-1565.

Change of Authorship Form

(Must be completed and signed by ALL authors)

Please check all that apply

__✓__ New author(s) have been added (in addition to this form, all new authors must complete the copyright transfer agreement and conflict of interest disclosure.

_____ Change in order of authorship.

_____ An author wishes to remove his/her name. An author's name may only be removed his/her own request and a letter signed by the author should accompany this form

Manuscript Number HYPE2019141

Manuscript Title Regional patterns and association between obesity and hypertension in Africa: E

Former Authorship

Please list ALL AUTHORS in the same order as the original submission. For more than 12, use an extra sheet.

Print Name		Print Name	
Name (1)	Michèle Ramsay	Name (7)	
Name (2)	Mayowa O. Owolabi	Name (8)	
Name (3)		Name (9)	
Name (4)		Name (10)	
Name (5)		Name (11)	
Name (6)		Name (12)	

New Authorship

All authors must sign below agreeing to the changes in authorship. The authorship order must reflect the authorship order of the manuscript.

Name		Signature	Date
Name (1)	Rufus Akinyemi		
Name (2)	Mulugeta Gebregziabher		
Name (3)	Fred Sarfo		
Name (4)	Kolawole Wahab		
Name (5)	Godfred Agongo		
Name (6)	Marianne Alberts	[signature]	
Name (7)	Stuart Ali		
Name (8)	Gershim Asiki		
Name (9)	Romuald Boua		
Name (10)	F Xavier Gómez-Olivé,		
Name (11)	Felistas Mashinya		
Name (12)	Lisa Micklesfield		

Please print form and fax to 214-706-1565.

Change of Authorship Form

(Must be completed and signed by ALL authors)

Please check all that apply

__✔__ New author(s) have been added (in addition to this form, all new authors must complete the copyright transfer agreement and conflict of interest disclosure.

_____Change in order of authorship.

_____An author wishes to remove his/her name. An author's name may only be removed his/her own request and a letter signed by the author should accompany this form

Manuscript Number HYPE2019141

Manuscript Title Regional patterns and association between obesity and hypertension in Africa: E

Former Authorship

Please list ALL AUTHORS in the same order as the original submission. For more than 12, use an extra sheet.

Print Name	Print Name
Name (1) Michèle Ramsay	Name (7)
Name (2) Mayowa O. Owolabi	Name (8)
Name (3)	Name (9)
Name (4)	Name (10)
Name (5)	Name (11)
Name (6)	Name (12)

New Authorship

All authors must sign below agreeing to the changes in authorship. The authorship order must reflect the authorship order of the manuscript.

Name	Signature	Date
Name (1) Rufus Akinyemi	Signature	Date
Name (2) Mulugeta Gebregziabher	Signature	Date
Name (3) Fred Sarfo	Signature	Date
Name (4) Kolawole Wahab	Signature	Date
Name (5) Godfred Agongo	Signature	Date
Name (6) Marianne Alberts	Signature	Date
Name (7) Stuart Ali	Signature	Date
Name (8) Gershim Asiki	Signature	Date
Name (9) Romuald Boua	Signature	Date
Name (10) F Xavier Gómez-Olivé,	Signature	Date
Name (11) Felistas Mashinya	Signature F. Mashinya	Date 24/01/2020
Name (12) Lisa Micklesfield	Signature	Date

Please print form and fax to 214-706-1565.

Change of Authorship Form

(Must be completed and signed by ALL authors)

Please check all that apply

_____New author(s) have been added (in addition to this form, all new authors must complete the copyright transfer agreement and conflict of interest disclosure.

_____Change in order of authorship.

_____An author wishes to remove his/her name. An author's name may only be removed his/her own request and a letter signed by the author should accompany this form

Manuscript Number HYPE2019141

Manuscript Title Regional patterns and association between obesity and hypertension in Africa: E

Former Authorship

Please list ALL AUTHORS in the same order as the original submission. For more than 12, use an extra sheet.

Print Name	Print Name
Name (1) Michèle Ramsay	Name (7)
Name (2) Mayowa O. Owolabi	Name (8)
Name (3)	Name (9)
Name (4)	Name (10)
Name (5)	Name (11)
Name (6)	Name (12)

New Authorship

All authors must sign below agreeing to the changes in authorship. The authorship order must reflect the authorship order of the manuscript.

Name	Signature	Date
Name (1) Rufus Akinyemi		
Name (2) Mulugeta Gebregziabher	M.Gebregziabher	
Name (3) Fred Sarfo		
Name (4) Kolawole Wahab		
Name (5) Godfred Agongo		
Name (6) Marianne Alberts		
Name (7) Stuart Ali		
Name (8) Gershim Asiki		
Name (9) Romuald Boua	BOUA Palwendé Romuald	24Jan 202[illegible]
Name (10) F Xavier Gómez-Olivé,		
Name (11) Felistas Mashinya		
Name (12) Lisa Micklesfield		

Please print form and fax to 214-706-1565.

Change of Authorship Form

(Must be completed and signed by ALL authors)

Please check all that apply

_____New author(s) have been added (in addition to this form, all new authors must complete the copyright transfer agreement and conflict of interest disclosure.

_____Change in order of authorship.

_____An author wishes to remove his/her name. An author's name may only be removed his/her own request and a letter signed by the author should accompany this form

Manuscript Number HYPE201914147-T1

Manuscript Title Regional patterns and association between obesity and hypertension in Africa: Evidence from the H3Africa CHAIR study

Former Authorship

Please list ALL AUTHORS in the same order as the original submission. For more than 12, use an extra sheet.

**Print Name**		**Print Name**	
Name (1)	Michèle Ramsay	Name (7)	
Name (2)	Mayowa O. Owolabi	Name (8)	
Name (3)		Name (9)	
Name (4)		Name (10)	
Name (5)		Name (11)	
Name (6)		Name (12)	

New Authorship

All authors must sign below agreeing to the changes in authorship. The authorship order must reflect the authorship order of the manuscript.

Name		Signature	Date
Name (1)	Rufus Akinyemi	RufusAkinyemi	25/01/20
Name (2)	Mulugeta Gebregziabher		
Name (3)	Fred Sarfo		
Name (4)	Kolawole Wahab	K.Wahab	28/01/20
Name (5)	Godfred Agongo		
Name (6)	Marianne Alberts		
Name (7)	Stuart Ali	StuartAli	27/01/2020
Name (8)	Gershim Asiki		
Name (9)	Romuald Boua		
Name (10)	F Xavier Gómez-Olivé,	[signature]	24 January 2020
Name (11)	Felistas Mashinya		
Name (12)	Lisa Micklesfield		

Please print form and fax to 214-706-1565.

Change of Authorship Form

(Must be completed and signed by ALL authors)

Please check all that apply

_____New author(s) have been added (in addition to this form, all new authors must complete the copyright transfer agreement and conflict of interest disclosure.

_____Change in order of authorship.

_____An author wishes to remove his/her name. An author's name may only be removed his/her own request and a letter signed by the author should accompany this form

Manuscript Number HYPE2019141

Manuscript Title Regional patterns and association between obesity and hypertension in Africa: I

Former Authorship

Please list ALL AUTHORS in the same order as the original submission. For more than 12, use an extra sheet.

Print Name	Print Name
Name (1) ____________	Name (7) ____________
Name (2) ____________	Name (8) ____________
Name (3) ____________	Name (9) ____________
Name (4) ____________	Name (10) ____________
Name (5) ____________	Name (11) ____________
Name (6) ____________	Name (12) ____________

New Authorship

All authors must sign below agreeing to the changes in authorship. The authorship order must reflect the authorship order of the manuscript.

Name	Signature	Date
Name (1) Shukri Mohamed	Signature	Date
Name (2) Engelbert Nonterah	Signature	Date
Name (3) Shane Norris	Signature	Date
Name (4) Hermann Sorgho	Signature H Sorgho	Date 24/01/2020
Name (5) Stephen Tollman,	Signature	Date
Name (6) Rulan Parekh	Signature	Date
Name (7) Chisala Chisala	Signature	Date
Name (8) Kenneth Ekoru	Signature	Date
Name (9) Salina P. Waddy	Signature	Date
Name (10) Emmanuel Peprah	Signature	Date
Name (11) George A. Mensah	Signature	Date
Name (12) Ken Wiley	Signature	Date

Please print form and fax to 214-706-1565.

Change of Authorship Form

(Must be completed and signed by ALL authors)

Please check all that apply

_____New author(s) have been added (in addition to this form, all new authors must complete the copyright transfer agreement and conflict of interest disclosure.

_____Change in order of authorship.

_____An author wishes to remove his/her name. An author's name may only be removed his/her own request and a letter signed by the author should accompany this form

Manuscript Number HYPE2019141

Manuscript Title Regional patterns and association between obesity and hypertension in Africa: E

Former Authorship

Please list ALL AUTHORS in the same order as the original submission. For more than 12, use an extra sheet.

Print Name		Print Name	
Name (1)		Name (7)	
Name (2)		Name (8)	
Name (3)		Name (9)	
Name (4)		Name (10)	
Name (5)		Name (11)	
Name (6)		Name (12)	

New Authorship

All authors must sign below agreeing to the changes in authorship. The authorship order must reflect the authorship order of the manuscript.

Name		Signature	Date
Name (1)	Shukri Mohamed	Shukri Mohamed	24/01/20
Name (2)	Engelbert Nonterah		
Name (3)	Shane Norris		
Name (4)	Hermann Sorgho		
Name (5)	Stephen Tollman,		
Name (6)	Rulan Parekh		
Name (7)	Chishala Chishala		
Name (8)	Kenneth Ekoru		
Name (9)	Salina P. Waddy		
Name (10)	Emmanuel Peprah		
Name (11)	George A. Mensah		
Name (12)	Ken Wiley	Kenneth L. Wiley Jr -S Digitally signed by Kenneth L. Wiley Jr -S Date: 2020.01.23 21:09:01 -05'00'	1/23/2020

Please print form and fax to 214-706-1565.

Change of Authorship Form

(Must be completed and signed by ALL authors)

Please check all that apply

_✓_ New author(s) have been added (in addition to this form, all new authors must complete the copyright transfer agreement and conflict of interest disclosure.

_____Change in order of authorship.

_____An author wishes to remove his/her name. An author's name may only be removed his/her own request and a letter signed by the author should accompany this form

Manuscript Number HYPE2019141

Manuscript Title Regional patterns and association between obesity and hypertension in Africa: E

Former Authorship

Please list ALL AUTHORS in the same order as the original submission. For more than 12, use an extra sheet.

Print Name	Print Name
Name (1) ______	Name (7) ______
Name (2) ______	Name (8) ______
Name (3) ______	Name (9) ______
Name (4) ______	Name (10) ______
Name (5) ______	Name (11) ______
Name (6) ______	Name (12) ______

New Authorship

All authors must sign below agreeing to the changes in authorship. The authorship order must reflect the authorship order of the manuscript.

Name	Signature	Date
Name (1) Shukri Mohamed	Text	
Name (2) Engelbert Nonterah		
Name (3) Shane Norris		
Name (4) Hermann Sorgho		
Name (5) Stephen Tollman,	S Tollman	24. 01. 2020
Name (6) Rulan Parekh		
Name (7) Chisala Chisala		
Name (8) Kenneth Ekoru		
Name (9) Salina P. Waddy		
Name (10) Emmanuel Peprah		
Name (11) George A. Mensah		
Name (12) Ken Wiley		

Please print form and fax to 214-706-1565.

Change of Authorship Form

(Must be completed and signed by ALL authors)

Please check all that apply

_____New author(s) have been added (in addition to this form, all new authors must complete the copyright transfer agreement and conflict of interest disclosure.

_____Change in order of authorship.

_____An author wishes to remove his/her name. An author's name may only be removed his/her own request and a letter signed by the author should accompany this form

Manuscript Number HYPE2019141

Manuscript Title Regional patterns and association between obesity and hypertension in Africa: E

Former Authorship

Please list ALL AUTHORS in the same order as the original submission. For more than 12, use an extra sheet.

Print Name	Print Name
Name (1) ______	Name (7) ______
Name (2) ______	Name (8) ______
Name (3) ______	Name (9) ______
Name (4) ______	Name (10) ______
Name (5) ______	Name (11) ______
Name (6) ______	Name (12) ______

New Authorship

All authors must sign below agreeing to the changes in authorship. The authorship order must reflect the authorship order of the manuscript.

Name		Signature	Date
Name (1)	Shukri Mohamed		
Name (2)	Engelbert Nonterah		
Name (3)	Shane Norris		
Name (4)	Hermann Sorgho		
Name (5)	Stephen Tollman,		
Name (6)	Rulan Parekh		
Name (7)	Chisala Chisala		
Name (8)	Kenneth Ekoru		
Name (9)	Salina P. Waddy		
Name (10)	Emmanuel Peprah		
Name (11)	George A. Mensah	George A. Mensah -S Digitally signed by George A. Mensah -S Date: 2020.01.23 23:10:41 -05'00'	01/23/202[illegible]
Name (12)	Ken Wiley		

Please print form and fax to 214-706-1565.

Change of Authorship Form

(Must be completed and signed by ALL authors)

Please check all that apply

_____New author(s) have been added (in addition to this form, all new authors must complete the copyright transfer agreement and conflict of interest disclosure.

_____Change in order of authorship.

_____An author wishes to remove his/her name. An author's name may only be removed his/her own request and a letter signed by the author should accompany this form

Manuscript Number HYPE2019141

Manuscript Title Regional patterns and association between obesity and hypertension in Africa: E

Former Authorship

Please list ALL AUTHORS in the same order as the original submission. For more than 12, use an extra sheet.

Print Name	Print Name
Name (1) ____________	Name (7) ____________
Name (2) ____________	Name (8) ____________
Name (3) ____________	Name (9) ____________
Name (4) ____________	Name (10) ____________
Name (5) ____________	Name (11) ____________
Name (6) ____________	Name (12) ____________

New Authorship

All authors must sign below agreeing to the changes in authorship. The authorship order must reflect the authorship order of the manuscript.

Name	Signature	Date
Name (1) Shukri Mohamed	Signature	Date
Name (2) Engelbert Nonterah	Signature	Date
Name (3) Shane Norris	Signature	Date
Name (4) Hermann Sorgho	Signature	Date
Name (5) Stephen Tollman,	Signature	Date
Name (6) Rulan Parekh	Signature	Date
Name (7) Chisala Chisala	Signature	Date
Name (8) Kenneth Ekoru	Signature	Date
Name (9) Salina P. Waddy	Signature	Date
Name (10) Emmanuel Peprah	Signature EK Peprah	Date 1-24-2020
Name (11) George A. Mensah	Signature	Date
Name (12) Ken Wiley	Signature	Date

Please print form and fax to 214-706-1565.

Change of Authorship Form

(Must be completed and signed by ALL authors)

Please check all that apply

_____New author(s) have been added (in addition to this form, all new authors must complete the copyright transfer agreement and conflict of interest disclosure.

_____Change in order of authorship.

_____An author wishes to remove his/her name. An author's name may only be removed his/her own request and a letter signed by the author should accompany this form

Manuscript Number HYPE201914147-T1

Manuscript Title Regional patterns and association between obesity and hypertension in Africa: Evidence from the H3Africa CHAIR study

Former Authorship

Please list ALL AUTHORS in the same order as the original submission. For more than 12, use an extra sheet.

Print Name	Print Name
Name (1) ______	Name (7) ______
Name (2) ______	Name (8) ______
Name (3) ______	Name (9) ______
Name (4) ______	Name (10) ______
Name (5) ______	Name (11) ______
Name (6) ______	Name (12) ______

New Authorship

All authors must sign below agreeing to the changes in authorship. The authorship order must reflect the authorship order of the manuscript.

	Name	Signature	Date
Name (1)	Shukri Mohamed		
Name (2)	Engelbert Nonterah		
Name (3)	Shane Norris		
Name (4)	Hermann Sorgho		
Name (5)	Stephen Tollman,		
Name (6)	Rulan Parekh		
Name (7)	Chisala Chisala		
Name (8)	Kenneth Ekoru	[signature]	01-23-20
Name (9)	Salina P. Waddy		
Name (10)	Emmanuel Peprah		
Name (11)	George A. Mensah		
Name (12)	Ken Wiley		

Please print form and fax to 214-706-1565.

Change of Authorship Form

(Must be completed and signed by ALL authors)

Please check all that apply

_____New author(s) have been added (in addition to this form, all new authors must complete the copyright transfer agreement and conflict of interest disclosure.

_____Change in order of authorship.

_____An author wishes to remove his/her name. An author's name may only be removed his/her own request and a letter signed by the author should accompany this form

Manuscript Number HYPE2019141

Manuscript Title Regional patterns and association between obesity and hypertension in Africa: E

Former Authorship

Please list ALL AUTHORS in the same order as the original submission. For more than 12, use an extra sheet.

Print Name	Print Name
Name (1) ________	Name (7) ________
Name (2) ________	Name (8) ________
Name (3) ________	Name (9) ________
Name (4) ________	Name (10) ________
Name (5) ________	Name (11) ________
Name (6) ________	Name (12) ________

New Authorship

All authors must sign below agreeing to the changes in authorship. The authorship order must reflect the authorship order of the manuscript.

Name	Signature	Date
Name (1) Shukri Mohamed	Signature ________	Date________
Name (2) Engelbert Nonterah	Signature ________	Date________
Name (3) Shane Norris	Signature ________	Date________
Name (4) Hermann Sorgho	Signature ________	Date________
Name (5) Stephen Tollman,	Signature ________	Date________
Name (6) Rulan Parekh	Signature ________	Date________
Name (7) Chisala Chisala	Signature ________	Date________
Name (8) Kenneth Ekoru	Signature ________	Date________
Name (9) Salina P. Waddy	Signature *Salina Waddy*	Date 01/23/2020
Name (10) Emmanuel Peprah	Signature ________	Date________
Name (11) George A. Mensah	Signature ________	Date________
Name (12) Ken Wiley	Signature ________	Date________

Please print form and fax to 214-706-1565.

Change of Authorship Form

(Must be completed and signed by ALL authors)

Please check all that apply

_____New author(s) have been added (in addition to this form, all new authors must complete the copyright transfer agreement and conflict of interest disclosure.

_____Change in order of authorship.

_____An author wishes to remove his/her name. An author's name may only be removed his/her own request and a letter signed by the author should accompany this form

Manuscript Number HYPE201914147-T1

Manuscript Title Regional patterns and association between obesity and hypertension in Africa: Evidence from the H3Africa CHAIR study

Former Authorship

Please list ALL AUTHORS in the same order as the original submission. For more than 12, use an extra sheet.

**Print Name**		**Print Name**	
Name (1)		Name (7)	
Name (2)		Name (8)	
Name (3)		Name (9)	
Name (4)		Name (10)	
Name (5)		Name (11)	
Name (6)		Name (12)	

New Authorship

All authors must sign below agreeing to the changes in authorship. The authorship order must reflect the authorship order of the manuscript.

	Name		Signature		Date
Name (1)	Shukri Mohamed	Signature		Date	
Name (2)	Engelbert A Nonterah	Signature	[illegible]	Date	24/01/2020
Name (3)	Shane Norris	Signature	Shane Norris	Date	24/01/20
Name (4)	Hermann Sorgho	Signature		Date	
Name (5)	Stephen Tollman,	Signature		Date	
Name (6)	Rulan Parekh	Signature	R. Parekh	Date	25/01/2020
Name (7)	Chisala Chisala	Signature	C.Chishala	Date	28/01/20
Name (8)	Kenneth Ekoru	Signature		Date	
Name (9)	Salina P. Waddy	Signature		Date	
Name (10)	Emmanuel Peprah	Signature		Date	
Name (11)	George A. Mensah	Signature		Date	
Name (12)	Ken Wiley	Signature		Date	

Please print form and fax to 214-706-1565.

Change of Authorship Form

(Must be completed and signed by ALL authors)

Please check all that apply

_____New author(s) have been added (in addition to this form, all new authors must complete the copyright transfer agreement and conflict of interest disclosure.

_____Change in order of authorship.

_____An author wishes to remove his/her name. An author's name may only be removed his/her own request and a letter signed by the author should accompany this form

Manuscript Number HYPE2019141

Manuscript Title Regional patterns and association between obesity and hypertension in Africa: E

Former Authorship

Please list ALL AUTHORS in the same order as the original submission. For more than 12, use an extra sheet.

**Print Name**	**Print Name**
Name (1) ____	Name (7) ____
Name (2) ____	Name (8) ____
Name (3) ____	Name (9) ____
Name (4) ____	Name (10) ____
Name (5) ____	Name (11) ____
Name (6) ____	Name (12) ____

New Authorship

All authors must sign below agreeing to the changes in authorship. The authorship order must reflect the authorship order of the manuscript.

Name (1) Jennifer Troyer	Signature ____	Date____
Name (2) Michèle Ramsay,	Signature [signature]	Date 25Jan2020
Name (3) Mayowa O. Owolabi	Signature ____	Date____
Name (4) ____	Signature ____	Date____
Name (5) ____	Signature ____	Date____
Name (6) ____	Signature ____	Date____
Name (7) ____	Signature ____	Date____
Name (8) ____	Signature ____	Date____
Name (9) ____	Signature ____	Date____
Name (10) ____	Signature ____	Date____
Name (11) ____	Signature ____	Date____
Name (12) ____	Signature ____	Date____

Please print form and fax to 214-706-1565.

Change of Authorship Form

(Must be completed and signed by ALL authors)

Please check all that apply

_____New author(s) have been added (in addition to this form, all new authors must complete the copyright transfer agreement and conflict of interest disclosure.

_____Change in order of authorship.

_____An author wishes to remove his/her name. An author's name may only be removed his/her own request and a letter signed by the author should accompany this form

Manuscript Number HYPE2019141

Manuscript Title Regional patterns and association between obesity and hypertension in Africa: E

Former Authorship

Please list ALL AUTHORS in the same order as the original submission. For more than 12, use an extra sheet.

**Print Name**	**Print Name**
Name (1) ______	Name (7) ______
Name (2) ______	Name (8) ______
Name (3) ______	Name (9) ______
Name (4) ______	Name (10) ______
Name (5) ______	Name (11) ______
Name (6) ______	Name (12) ______

New Authorship

All authors must sign below agreeing to the changes in authorship. The authorship order must reflect the authorship order of the manuscript.

Name	Signature	Date
Name (1) Jennifer Troyer	Signature . Jennifer Troyer	Date
Name (2) Michèle Ramsay,	Signature	Date
Name (3) Mayowa O. Owolabi	Signature	Date
Name (4)	Signature	Date
Name (5)	Signature	Date
Name (6)	Signature	Date
Name (7)	Signature	Date
Name (8)	Signature	Date
Name (9)	Signature	Date
Name (10)	Signature	Date
Name (11)	Signature	Date
Name (12)	Signature	Date

Please print form and fax to 214-706-1565.

Change of Authorship Form

(Must be completed and signed by ALL authors)

Please check all that apply

_____New author(s) have been added (in addition to this form, all new authors must complete the copyright transfer agreement and conflict of interest disclosure.

_____Change in order of authorship.

_____An author wishes to remove his/her name. An author's name may only be removed his/her own request and a letter signed by the author should accompany this form

Manuscript Number HYPE2019141

Manuscript Title Regional patterns and association between obesity and hypertension in Africa: E

Former Authorship

Please list ALL AUTHORS in the same order as the original submission. For more than 12, use an extra sheet.

**Print Name**	**Print Name**
Name (1) ________	Name (7) ________
Name (2) ________	Name (8) ________
Name (3) ________	Name (9) ________
Name (4) ________	Name (10) ________
Name (5) ________	Name (11) ________
Name (6) ________	Name (12) ________

New Authorship

All authors must sign below agreeing to the changes in authorship. The authorship order must reflect the authorship order of the manuscript.

Name		Signature	Date
Name (1)	Jennifer Troyer	Signature	Date
Name (2)	Michèle Ramsay,	Signature	Date
Name (3)	Mayowa O. Owolabi	Signature *Mayowa Owolabi*	Date 23010202C
Name (4)		Signature	Date
Name (5)		Signature	Date
Name (6)		Signature	Date
Name (7)		Signature	Date
Name (8)		Signature	Date
Name (9)		Signature	Date
Name (10)		Signature	Date
Name (11)		Signature	Date
Name (12)		Signature	Date

Please print form and fax to 214-706-1565.
